# The length of the G1 phase is an essential determinant of H3K27me3 landscapes across diverse cell types

**DOI:** 10.1371/journal.pbio.3003119

**Published:** 2025-04-17

**Authors:** Abby Trouth, Kameswaran Ravichandran, Philip R. Gafken, Sara Martire, Gabriel E. Boyle, Giovana M. B. Veronezi, Van La, Stephanie J. Namciu, Laura A. Banaszynski, Jay F. Sarthy, Srinivas Ramachandran

**Affiliations:** 1 Department of Biochemistry and Molecular Genetics, University of Colorado Anschutz Medical Campus, Aurora, Colorado, United States of America; 2 Proteomics and Metabolomics Shared Resource, Fred Hutchinson Cancer Center, Seattle, Washington, United States of America; 3 Cecil H. and Ida Green Center for Reproductive Biology Sciences, UT Southwestern Medical Center, Dallas, Texas, United States of America; 4 Ben Towne Center for Childhood Cancer Research, Seattle Children’s Research Institute, Seattle, Washington, United States of America; 5 RNA Bioscience Initiative, University of Colorado Anschutz Medical Campus, Aurora, Colorado, United States of America; National Cancer Institute, UNITED STATES OF AMERICA

## Abstract

Stem cells have lower facultative heterochromatin as defined by trimethylation of histone H3 lysine 27 (H3K27me3) compared to differentiated cells. However, the mechanisms underlying these differential H3K27me3 levels remain elusive. Because H3K27me3 levels are diluted 2-fold in every round of replication and then restored through the rest of the cell cycle, we reasoned that the cell cycle length could be a key regulator of total H3K27me3 levels. Here, we propose that a short G1 phase restricts H3K27me3 levels in stem cells. To test this model, we determined changes to H3K27me3 levels in mouse embryonic stem cells (mESCs) globally and at specific loci upon G1 phase lengthening – accomplished by thymidine block or growth in the absence of serum (with the “2i medium”). H3K27me3 levels in mESCs increase with G1 arrest when grown in serum and in 2i medium. Additionally, we observed via CUT&RUN and ChIP-seq that regions that gain H3K27me3 in G1 arrest and 2i media overlap, supporting our model of G1 length as a critical regulator of the stem cell epigenome. Furthermore, we demonstrate the inverse effect – that G1 shortening in differentiated human HEK293 cells results in a loss of H3K27me3 levels. Finally, in human tumor cells with extreme H3K27me3 loss, lengthening of the G1 phase leads to H3K27me3 recovery despite the presence of the dominant negative, sub-stoichiometric H3K27M mutation. Our results indicate that G1 length is an essential determinant of H3K27me3 landscapes across diverse cell types.

## Abstract 2

Stem cells have lower levels of facultative heterochromatin, defined by deposition of the H3K27me3 histone mark, compared to differentiated cells, but why is this? By artificially lengthening the G1 phase, this study shows that G1 length is an essential determinant of H3K27me3 landscape across diverse cell types.

## Introduction

Pluripotent stem cells must balance differentiation potential with their cellular identity [1]. Maintenance of this cellular identity across multiple divisions is accomplished in part by the inheritance of parental chromatin states [2]. These states include the trimethylation of histone H3 lysine K27 (H3K27me3) deposited by polycomb repressive complex 2 (PRC2). PRC2 deposits H3K27me3 across large chromatin domains that contribute to cell-type specific silencing [3]. As a result, the global distribution of H3K27me3 is also cell-type specific [4]. Pluripotent stem cells have unique features in terms of H3K27me3 [5]: it is present at lineage specifying genes at low levels, leading to speculation that maintaining levels of silencing more amenable to change upon activation might be enough to maintain stem cell pluripotency while at the same time providing a lower barrier for differentiation. Stem cells must remain on the cusp of differentiation, requiring limited and reversible silencing in part through their facultative heterochromatin structures. The mechanisms stem cells utilize to maintain low levels of H3K27me3 remain unclear, though a potential candidate arises when comparing how different cell types proceed through the cell cycle.

The cell cycle – consisting of the G1, S, and G2 phases followed by mitosis – is traversed to produce two identical daughter cells with the same chromatin landscape as the parent cell from which they arose. However, the DNA replication within the S phase challenges the levels of pre-existing epigenetic modifications like H3K27me3, as modified parental histones are divided amongst the two daughter strands in addition to the deposition of new, unmodified histones [6]. This dilution to approximately half the original H3K27me3 levels must be replenished before the subsequent replication to avoid a loss of silencing across multiple divisions. While every cell must overcome this dilution obstacle, cell cycle dynamics are highly heterogeneous across cell types, with notable differences between stem and differentiated cells. Stem cells speed through their cell cycle, with many examples where they forgo checkpoints or whole cycle phases. In the early embryonic stages of *Drosophila*, the cycle is 8 min long and composed of only the S phase and mitosis [7]. Serum-grown mouse embryonic stem cells (mESCs) divide in approximately 11 h [8], the lack of a G1 checkpoint ultimately shortening the phase length [9]. The retention of the G1 checkpoint is observed in mESCs grown without serum (in the presence of two kinase inhibitors, PD0325901 and CHIR99021, the “2i medium” [10]), leading to an extended G1 in comparison. While 2i mESCs possess higher global levels of H3K27me3 compared to serum-grown cells, both growth conditions result in low levels of H3K27me3 relative to differentiated cells [11]. Previous studies have reported changes in the levels of key PRC2 subunits – including the catalytic EZH1/EZH2 subunits – when comparing pluripotent and differentiated cell types [3,12] as well as serum-grown versus 2i mESCs [13], leading us to speculate that both the time spent in the cell cycle and varying levels of PRC2 activity could impact the global levels of H3K27me3 and heterochromatin in general.

We propose that the faster cell cycles observed in embryonic stem cells restrict H3K27me3 levels to maintain heterochromatin domain structures potentially needed for a balanced pluripotent state. To test this model, we determined changes to H3K27me3 levels in mESCs both globally and at specific loci upon G1 phase lengthening – accomplished by thymidine block or growth in the absence of serum (“2i medium” [10]). H3K27me3 levels in mESCs increase with G1 arrest when grown in serum and are higher in 2i medium. Additionally, we observed via CUT&RUN and ChIP-seq that regions that gain H3K27me3 in G1 arrest and 2i media overlap, supporting our model of G1 length as a critical regulator in the stem cell epigenome. Furthermore, we demonstrate the inverse effect – that G1 shortening in differentiated cells results in a loss of H3K27me3 global levels via domain shrinking. Finally, in tumor cells with extreme H3K27me3 loss, lengthening of the G1 phase leads to H3K27me3 recovery despite the presence of the dominant negative, sub-stoichiometric H3K27M mutation. Our results indicate that G1 length is an essential determinant of facultative heterochromatin landscapes across diverse cell types.

## Results

### Lengthening G1 increases global levels of H3K27me3 in mESC

**Fig 1:**
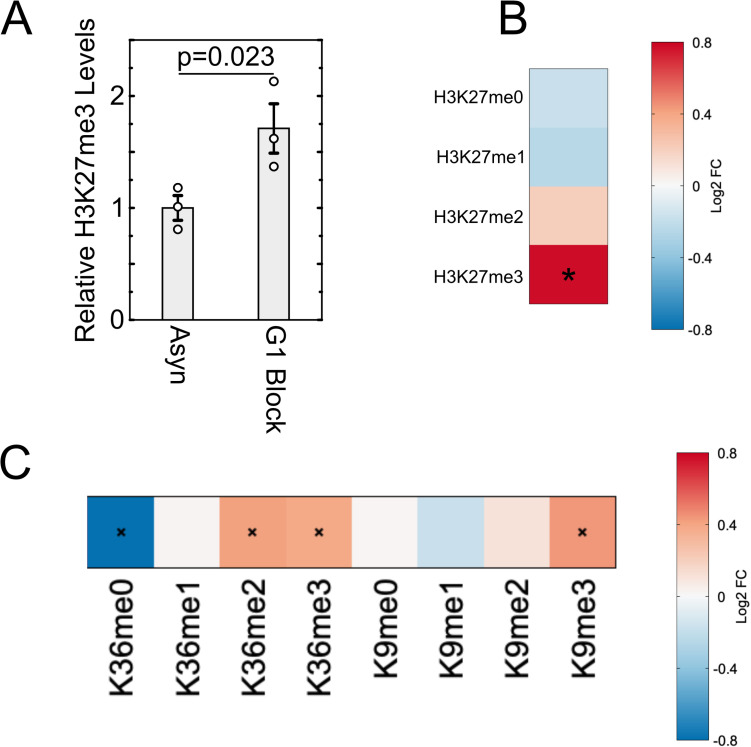
Global H3K27me3 levels in serum/LIF-grown mESCs increase with G1/S arrest. (A). Change in relative H3K27me3 levels determined via mass spectrometry across three replicates for cells that underwent double thymidine block with the second block lasting 20 h. (B). Heat map of average fold change across three replicates in H3K27 methylation states in G1 arrested cells versus asynchronous cells. (C). Heat map of fold change in methylation states for H3K36 and H3K9 after a 20-h G1/S arrest for average values across the three replicates. Values for each methylation state were normalized to values obtained from asynchronous cells. Asterisk (*) indicates *p* < 0.05, *p*-values calculated using Student’s *t* test with a one-tailed distribution. The data underlying this figure can be found in S1 Data.

We hypothesized that the low level of H3K27me3 present in serum-grown mESCs is influenced by the short G1 phase in these cells. To test this hypothesis, we analyzed histones by mass spectrometry after a double thymidine block to induce a G1 arrest, with a second block of 20 h. We determined that our mESC cell line doubles as expected [8], in approximately 10 h with 2–4 h spent in the G1 phase (S1 Fig). Thus, a 20 h block represents a significant increase in G1 phase. We then purified histones by acid extraction and analyzed them by mass spectrometry (MS) to reveal all states of H3K27 methylation and quantify the change in H3K27me3 due to lengthened G1. We observed an increase in H3K27me3 in arrested cells accompanied by a decrease in H3K27me0/1 (Fig 1A and 1B). Thus, G1 lengthening results in a progression of methylation, converting unmethylated H3K27 and H3K27me1 towards H3K27me2 and H3K27me3. Since MS profiles many other modification states of histone tails in the same experiment, we next looked at H3K36 and H3K9 methylation and found a pattern identical to H3K27me3 (Fig 1C). In summary, histone methylation states correlated with G1 length in mESCs, with H3K27me3 showing the most substantial increase upon lengthening G1. This shift towards the trimethylated state and depletion of unmethylated H3K27 residues suggests that global H3K27 methylation states in mESCs are influenced by G1 length.

### G1 extension results in domain spreading and de novo domain formation

**Fig 2:**
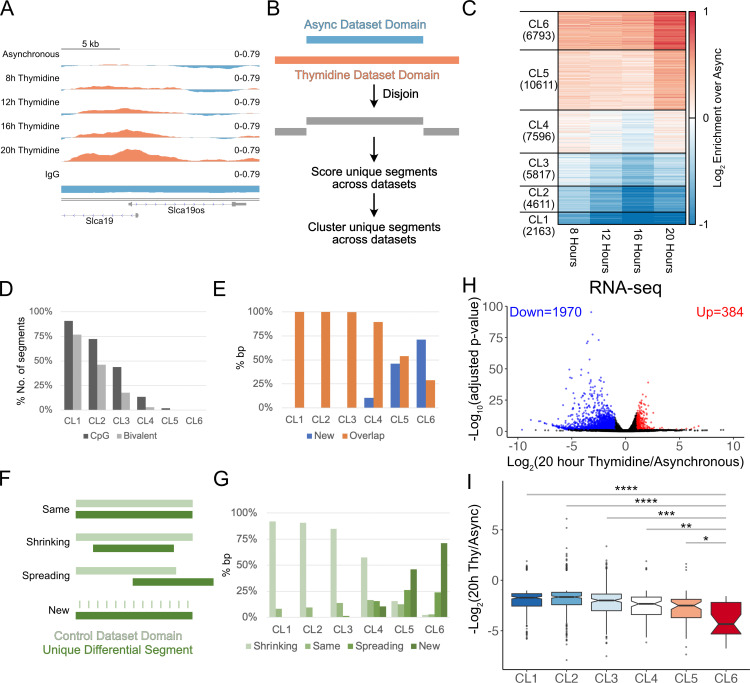
Locus-specific changes in H3K27me3 upon G1/S arrest. (A). An example of a genomic region showing progressive gain in H3K27me3 upon thymidine treatment. The genomic snapshot was created using IGV, setting the midpoint of the data range as the lower cut-off used in calling domains. Thus, data above midpoint (red) would belong to domains, whereas data below midpoint (blue) would be outside domains. (B). Schematic detailing how non-overlapping segments are defined by comparing H3K27me3 domains for asynchronous and thymidine-treated mESCs. (C). Heatmap of H3K27me3 CUT&RUN enrichment from three replicates combined at four time points of G1/S arrest after performing *k*-mean clustering with 6 clusters. Each horizontal line of the heatmap represents a unique segment determined by Disjoin. (D). Percentage of the number of segments in each of the 6 clusters containing CpG islands or bivalent promoters. (E). Percentage of bp covered by segments belonging to each of the 6 clusters defined in **(C)** that overlaps with H3K27me3 domains present in asynchronous mESCs. (F). Schematic detailing four domain changes observed after thymidine arrest. Unique segments from thymidine datasets were classified as “same” when maintaining the same boundaries as an asynchronous domain, “shrinking” when one or more boundaries retract, “spreading” when one or more boundaries extend past those of the asynchronous domain, and “new” for domains not present in asynchronous cells. (G). Percentage of bp covered by segments belonging to each of the four categories of domain changes (shrinking, same, spreading, and new) observed in the 6 clusters defined in (C). (H). Volcano plot where the Log2(Fold Change) in RNA for 20 h Thymidine vs. asynchronous is plotted against -log10(FDR) for genes. Genes with Log2(Fold Change) >1 and FDR < 0.05 are marked in red, whereas genes with Log2(Fold Change) <-1 and FDR < 0.05 are marked in blue. (I). Log2(Fold Change) in RNA of significantly changing genes belonging to each cluster plotted as boxplots. *P*-values were calculated using Wilcoxon rank sum test and adjusted for multiple testing using Benjamini-Hochberg procedure. The data underlying Fig 2A can be found at accession GSE264214 in GEO, the data underlying Fig 2D, 2E, 2G–I can be found in S2 Data.

The global increase in H3K27me3 upon extension of G1 could result from three scenarios – (i) an increase in enrichment in existing H3K27me3 domains, (ii) spread beyond the boundaries of existing domains to form larger ones, or (iii) creation of new domains. To ask how the genome-wide distribution of H3K27me3 changed upon G1 extension, we performed CUT&RUN on H3K27me3 in triplicate after a single thymidine block for 8, 12, 16, and 20 h, with asynchronous cells as control. We observed several loci with H3K27me3 gain proportional to the length of thymidine block (Figs 2A and S2). To analyze these trends genome-wide, we first defined domains for each time point, then compared domain definitions across time points to identify unique, non-overlapping segments that would make up the superset of domains across time points (Fig 2B). Among the six clusters obtained by *k*-means clustering, clusters 5 and 6 featured a gain in H3K27me3 and accounted for most segments of H3K27me3 that changed upon the thymidine block (41% bp of segments, covering a total of approximately 87 million bp) (Fig 2C). Interestingly, clusters 1 and 2 exhibited a loss in H3K27me3, showing that, even though H3K27me3 goes up globally, some segments in the genome still lose H3K27me3. Thus, a more extended G1 phase rewires the genome-wide H3K27me3 landscape in mESCs.

PRC2 is known to act on two prominent, overlapping genomic features in mESCs [14]: CpG islands with low DNA methylation and bivalent promoters featuring both H3K27me3 and H3K4me3. Having clustered H3K27me3 domain changes in mESCs upon G1/S arrest, we next investigated the extent to which clusters overlapped with the two known markers of PRC2 activity. We observed a striking trend where the fraction of segments containing CpG islands/bivalent promoters monotonically decreased from clusters 1 to 6 (Fig 2D). Notably, the two clusters with the most significant gain in H3K27me3 – CL5/6 – show almost no overlap with CpG islands or bivalent promoters. As expected from the overlap of CL1/2 segments with bivalent promoters, genes within these segments are enriched for GO terms related to development, including cell fate commitment, embryonic organ development, neural, and cardiac development (S3 Fig). Genes within CL5 segments are enriched for immune genes, whereas genes within CL6 segments are enriched for cytoskeletal and protease genes (S4 Fig).

The lack of overlap between CpG islands/bivalent promoters and clusters 5 and 6 leads to two possibilities: (i) the observed H3K27me3 increase in longer G1 phases may result from PRC2 spreading away from CpG islands and bivalent promoters or (ii) the extended time in G1 allows for de novo nucleation at new sites that are not in proximity to these elements. To determine which mechanism is at play, we first calculated the coverage in bp of segments in each cluster that overlapped with H3K27me3 domains in asynchronous cells. In clusters 5 and 6, we observed new segments that did not intersect with domains in asynchronous cells, indicating de novo nucleation due to G1 extension (Fig 2E). The new segments contributed 46% and 71% in terms of bp-coverage to clusters 5 and 6, respectively. We next categorized the segments that do overlap with domains in asynchronous cells as “same”/”shrinking”/”spreading” (Fig 2F). Clusters 1, 2, and 3 mainly feature shrinking domains due to thymidine treatment, whereas clusters 5 and 6 comprise some spreading but are predominantly made up of new domains (Fig 2G). In summary, G1 extension results in more regions in the genome gaining H3K27me3 than losing it. The observed gain in H3K27me3 is partly facilitated by PRC2 spreading at previously defined domains. However, the modification is mainly gained by forming new domains that do not contain known PRC2 recruitment features. Our results suggest that extending the G1 phase allows weaker nucleation sites to develop as domains, pointing to G1 length as an essential determinant in the genome-wide H3K27me3 landscape.

### H3K27me3 gain due to G1 extension correlates with decreased RNA levels

To ask if there was any impact of changing H3K27me3 landscape on gene expression, we performed polyA-selected RNA-seq on triplicate samples of asynchronous mESCs and mESCs treated with thymidine for 20 h. Principal component analysis revealed a clear separation between asynchronous and thymidine block samples, while the replicates clustered together (S5A Fig). We next plotted the normalized counts for pluripotency and lineage markers to ask if thymidine block changes pluripotency state of our mESCs. The pluripotency markers were highly expressed in thymidine blocked cells, whereas the differentiation markers for the three lineages were all at low levels (at least 100-fold lower than pluripotency markers), confirming that pluripotent state was strongly maintained during thymidine block (S5B Fig). We next identified genes that had a significant change in levels (adjusted *p*-value < 0.05 and log_2_ fold change > |1|) in cells treated with thymidine compared to asynchronous cells. We observed 1970 genes to be significantly downregulated and 384 genes to be significantly upregulated due to thymidine block (Fig 2H). The much larger number of downregulated genes agrees with a global increase in heterochromatin due to G1 extension. GO enrichment analysis of upregulated genes revealed that GO terms relating to chromatin factors (including histones) and cell division are enriched, reflecting G1-S block. GO terms relating to DNA repair were not enriched in upregulated genes, supporting the idea that the H3K27me3 increases we observe may not be due to DNA repair response to thymidine treatment. We next plotted the log_2_ fold change for significantly changing genes in segments belonging to each of the six clusters (defined in Fig 2C) of H3K27me3 change due to extended G1. Significantly changing genes in all six clusters are predominantly downregulated, however, genes in clusters 5 and 6 have significantly lower log_2_ fold changes compared to the other clusters (Fig 2I). Thus, the extent of downregulation due to G1 extension correlates with the extent of H3K27me3 gain. In summary, gene expression changes point to silencing of new regions with H3K27me3 upon G1 extension.

### Weak PRC2 nucleation sites drive H3K27me3 gain due to G1 extension

**Fig 3:**
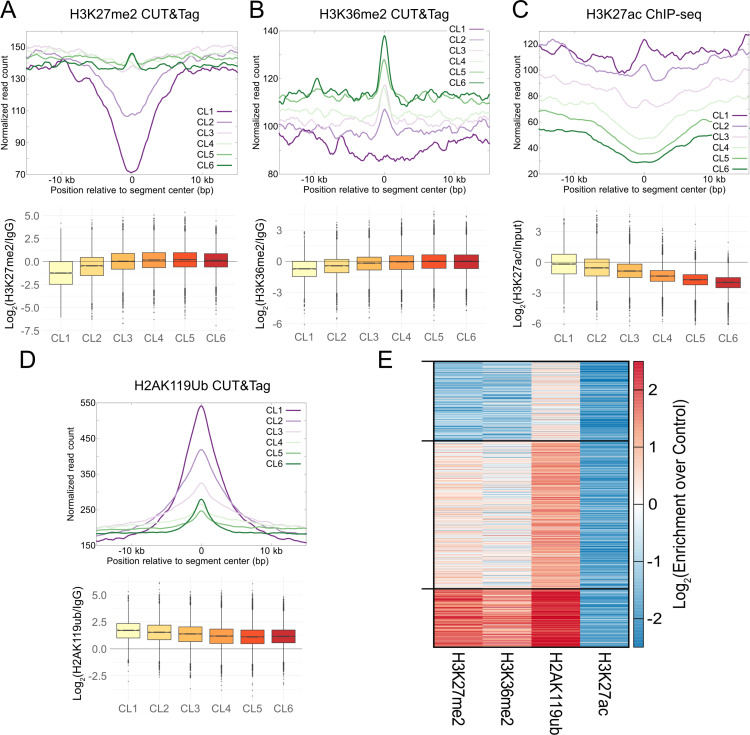
H3K27me3 gains due to longer G1 occur at weak nucleation sites. (A). Enrichment of H3K27me2 CUT&Tag normalized read count averaged over unique segments in each thymidine cluster defined in Fig 2, plotted relative to centers of the unique segments (top). The log_2_ ratio of the H3K27me2 normalized read count at each segment to the IgG normalized read count is plotted as a boxplot for each thymidine cluster (bottom). (B). Same as (**A**) for H3K36me2 CUT&Tag. (C) Same as **(A)** for H3K27ac ChIP-seq. (D). Same as **(A)** for H2AK119ub CUT&Tag. **(E)**. *k*-means clustering (*k* = 3) of log_2_ enrichment of H3K27me2, H3K36me2, H2AK119ub, and H3K27ac for segments in thymidine clusters 5 and 6 that gain H3K27me3 plotted as a heatmap. H3K27ac is depleted in all clusters, and enrichment of H3K27me2, H3K36me2, and H2AK119ub correlate with each other. The data underlying Fig 3A–E can be found in S3 Data.

We next asked what the chromatin features at steady state were for regions that gain H3K27me3 due to G1 extension using CUT&Tag. To validate CUT&Tag to be able to capture chromatin changes in these regions, we first performed H3K27me3 CUT&Tag in asynchronous mESCs and mESCs treated with thymidine for 20 h. The changes in H3K27me3 due to 20-h thymidine treatment as mapped by CUT&Tag had a strong correlation with the changes observed by CUT&RUN (S6 Fig). We then proceeded to perform CUT&Tag for other modifications. We found the enrichment of four histone post-translational modifications to follow a trend from clusters 1 to 6. H3K27me2 and H3K36me2 enrichment increased going from clusters 1 to 6 (Fig 3A and 3B), whereas H3K27ac enrichment decreased from clusters 1 to 6 (Fig 3C). Thus, clusters 5 and 6 could represent regions coated by H3K27me2 and H3K36me2 to silence cryptic enhancers [15–17]. H2AK119ub had a non-monotonic trend: it decreased from clusters 1 to 4 but did not decrease further in clusters 5 and 6 (Fig 3D, bottom). At the center of the unique segments, the H2AK119ub enrichment goes up for cluster 6 after going down from clusters 1–5. Thus, clusters 5 and 6 feature significant levels of H2AK119ub, pointing to the possibility of segments in these clusters harboring weak nucleation sites for PRC1 and PRC2. We next asked if these four marks define different classes of chromatin in clusters 5 and 6 or if their trends correlate. We clustered the enrichment of these four marks using the *k*-means method (*k* = 3) and observed a consistent trend across segments (Fig 3E). H3K27ac is uniformly low, and levels of H3K27me2, H3K36me2, and H2AK119ub correlate with each other. In summary, the sites that gain H3K27me3 due to extended G1 appear to be weak nucleation sites for PRC1 and PRC2, most probably driven by NSD1 [15]. We conclude they are weak nucleation sites due to an increasing trend for H3K27me2, opposite of the decreasing trend of H3K27me3 at steady state. Thus, PRC2 binds these regions and can modify H3K27 up to the dimethylation state but not efficiently to trimethylation due to short G1. When G1 is extended, there might be enough time for PRC2 to methylate these weak nucleation sites to the trimethylation level, leading to the observed significant increase in H3K27me3 with longer G1.

### Regions that newly acquire H3K27me3 upon G1 extension overlap with 2i-specific H3K27me3 domains

**Fig 4:**
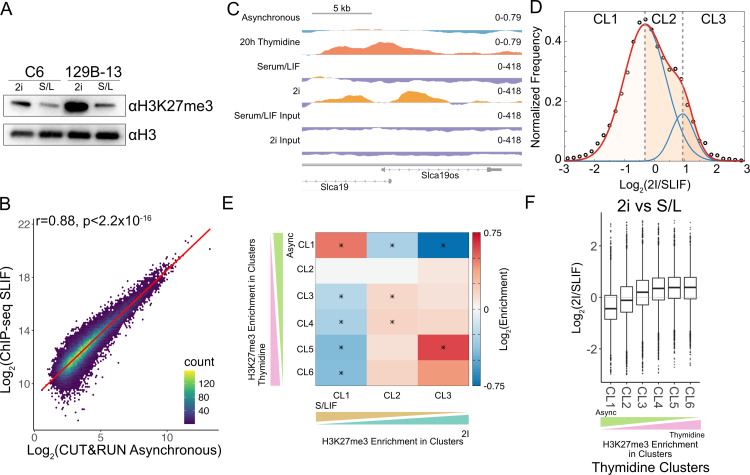
Regions with H3K27me3 gain in 2i-grown mESCs overlap with those in G1/S arrest of serum-grown mESC. (A). Immunoblot for H3K27me3 modification of two mESC cell lines grown in either serum/LIF (S/L or SLIF) media or 2i media. (B). Hexagonal binning of H3K27me3 enrichment at segments belonging to the six clusters defined in Fig 2C from ChIP-seq on the y-axis and CUT&RUN on the *x*-axis plotted using “geom_hex” in ggplot2. The line of best fit plotted using “geom_smooth” with method “lm” in ggplot2. The Pearson correlation coefficient and the associated *p*-value is indicated on the top left of the plot. (C). An example of a genomic region (same as shown in Fig 2A) showing gain in H3K27me3 upon thymidine treatment and in 2i compared to Serum/LIF. The genomic snapshot was created using IGV, setting the midpoint of the data range as the lower cut-off used in calling domains. Thus, data above midpoint (red/orange) would belong to domains, whereas data below midpoint (blue/purple) would be outside domains. (D). Distribution of the log_2_ ratio of H3K27me3 levels in 2i versus serum cells for unique domain segments shown as black circles. The distribution was fitted with the sum of two normal distributions (plotted in red). The individual distributions are plotted in blue. The shaded regions correspond to the three groups based on the log_2_ ratio: more H3K27me3 in SLIF cells (CL1, leftmost region), equivalent H3K27me3 in SLIF and 2i cells (CL2, center region), and regions with more H3K27me3 in 2i cells (CL3, rightmost region). (E). Extent of overlap of clusters of unique regions from G1/S block of serum grown cells and unique regions defined in (D). Asterisks denote statistical significance determined using hypergeometric test with multiple testing correction (corrected *p* < 0.05). (F). Log_2_ ratio of H3K27me3 enrichment of 2i mESC versus serum mESC calculated at unique segments defined in Fig 2. The data underlying Fig 4B, 4D, 4E, and 4F can be found in S4 Data, and data underlying Fig 4C available at accession GSE264216 in GEO.

Our experiments above were performed with mESCs grown in serum/LIF media. In these growth conditions, mESCs display a short G1 phase due to hyperphosphorylation of the Rb protein and subsequent lack of G1 checkpoints [9]. However, Rb is not hypophosphorylated in mESCs grown in 2i media, and the G1 checkpoint is functional, resulting in a longer G1 [9]. Thus, our previous results suggest that global H3K27me3 levels in 2i mESCs – which have been previously reported to be elevated – may in part be higher due to this change in G1 length compared to serum-grown cells. To confirm this higher global H3K27me3, we analyzed global H3K27me3 levels in mESCs from two sources, grown in 2i media or serum/LIF. We found higher levels of H3K27me3 in 2i mESCs compared to serum mESCs as described before [18] (Fig 4A).

Next, we performed H3K27me3 ChIP-seq in both conditions to compare H3K27me3 changes at the domain level between 2i and serum/LIF mESCs. The H3K27me3 ChIP-seq enrichment from serum/LIF mESCs correlated strongly with H3K27me3 CUT&RUN enrichment from our asynchronous mESCs grown in presence of serum, showing that we are capturing similar H3K27me3 profiles with both CUT&RUN and ChIP-seq (Fig 4B). Next, at the representative loci where we had observed increased H3K27me3 due to G1 extension by thymidine treatment, we also observed increased H3K27me3 in cells grown in 2i media compared to serum/LIF, suggesting that the G1 extension due to 2i media might mirror thymidine treatment in terms of locus-specific changes in H3K27me3 enrichment (Figs 4C and S7).

To extend this analyses genome-wide, we defined H3K27me3 domains using ChIP-seq datasets from 2i and serum/LIF cells and then identified unique segments when comparing 2i and serum/LIF domains. We observed a bimodal distribution in the ratio of H3K27me3 levels between these two conditions: the left-shifted distribution centered close to 0, indicating segments mostly similar between the two conditions; the right-shifted distribution centered close to 1, an average 2-fold increase in 2i compared to serum/LIF (Fig 4D). We defined 3 sets of regions – regions with higher H3K27me3 in serum/LIF (CL1), regions with equivalent H3K27me3 levels in both media (CL2), and regions with higher H3K27me3 levels in 2i (CL3) within the distribution (Fig 4D). If the length of the G1 phase was the underlying cause of the higher H3K27me3 observed in both 2i cells and thymidine-treated serum/LIF cells, then there should be significant overlap in regions that change in H3K27me3 levels between 2i cells and thymidine-treated serum/LIF cells. We overlapped clusters of segments in thymidine treatment with clusters from the 2i/serum/LIF comparison. We observe a significant enrichment between thymidine CL1 and 2i CL1, whereas a significant depletion between thymidine CL3-6 and 2i CL1. We also observe a significant enrichment between thymidine CL5 and 2i CL3 (Fig 4E). Thus, regions specific to serum/LIF cells compared to 2i cells overlap with regions that lose H3K27me3 upon G1 extension, and regions specific to 2i cells compared to serum/LIF gain H3K27me3 upon G1 extension. We can observe this also when we plot the log_2_ ratio of H3K27me3 in 2i compared to serum/LIF at thymidine clusters; we observed an increase going from thymidine CL1 to CL6 (Fig 4F). In other words, we can produce an H3K27me3 landscape genome-wide similar to ground-state pluripotent stem cells (2i) simply by extending G1 using a thymidine block.

### Shortening G1 in differentiated cells reduces H3K27me3 levels

**Fig 5:**
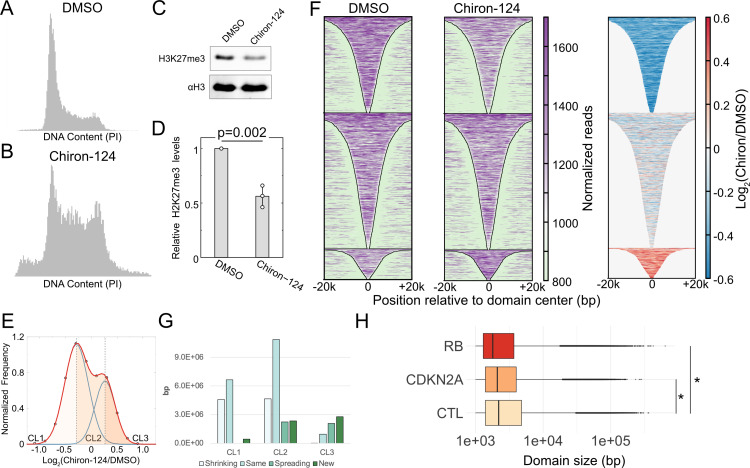
H3K27me3 domains in HEK293 cells change with accelerated cell cycle timing. (A). Flow cytometry analysis of DNA content using propidium iodide fluorescence for HEK293 cells that were treated with DMSO for 48 h. (B). Same as (A) HEK293 cells treated with Chiron-124 for 48 h. (C). Immunoblot for H3K27me3 and H3 on acid-extracted histones after 48-h treatment with Chiron-124. (D). Quantification of the modification levels normalized to DMSO-treatment. *p*-value calculated using two-tailed, homoscedastic student *t* test. (E). Distribution of the log_2_ ratio of H3K27me3 levels in Chiron-124 treatment compared to the DMSO treatment for unique domain segments. H3K27me3 levels were determined from the combined reads of two replicates for each condition. Data points are shown as black outlined circles; the sum of the two normal distributions is plotted as red; individual normal distributions are plotted as blue lines. The shaded regions correspond to the three groups based on the log_2_ ratio: loss of H3K27me3 upon Chiron-124 treatment (CL1, leftmost region), equivalent H3K27me3 in Chiron-124 and DMSO treatment (CL2, center region), and regions with more H3K27me3 in Chiron-124 treatment (CL3, rightmost region). (F). Heatmap of H3K27me3 enrichment at domains defined in DMSO treated cells for DMSO treatment (left) and Chiron-124 treatment (middle), and the log_2_ ratio of H3K27me3 within domains for Chiron-124 over DMSO (right). (G). Number of bp covered by segments belonging to each of the four categories of domain changes (shrinking, same, spreading, and new) observed in the three clusters shown in the bimodal distribution of H3K27me3 for Chiron-124 versus DMSO cells. (H). Distribution of H3K27me3 domain sizes across ENCODE cell lines with inactivated Rb or CDKN2A deletion or neither. Domain sizes were determined from SEGWAY annotations from ENCODE. *p < 2.2e–16 by Wilcoxon rank sum test. The data underlying Fig 5D–G can be found in S5 Data, with flow cytometry data for Fig 5A and B found in the flow repository public repository at accession FR-FCM-Z96P.

The global gain and locus-specific changes in H3K27me3 observed in serum/LIF-grown mESCs upon G1 phase extension illustrate a fundamental connection between the short G1 length and low heterochromatin levels in stem cells. We next explored if this principle holds across cell types by asking if a differentiated cell would dilute H3K27me3 levels when forced to proceed through a short G1 phase. To shorten G1 in differentiated cells, we treated HEK293 cells with the Chk1 inhibitor Chiron-124 [19] for 48 h – approximately the time for unperturbed cells to proceed through two divisions. After treatment, cells were stained with propidium iodide for cell cycle analysis by flow cytometry. We observed that most cells treated with DMSO were in G1 as expected, consistent with a long G1 phase (Fig 5A). In contrast and as expected, cells treated with Chiron-124 show a much more even distribution between all cell cycle phases as the inhibitor removes the G1 checkpoint (Fig 5B). We next determined the global H3K27me3 changes after Chiron-124 treatment. We purified histones by acid extraction and profiled H3K27me3 and H3 in HEK293 cells treated with DMSO or Chiron-124 for 48 h by immunoblotting (Fig 5C and 5D). After 48 h of Chiron-124 treatment, the cells possess approximately half of the global levels of H3K27me3 relative to H3 compared to the control DMSO-treated cells. This G1 phase acceleration was repeated in another system of cells with a longer G1 phase – the previously mentioned 2i mESCs. We treated 2i mESCs for 20 h with DMSO or Chiron-124, followed by immunoblotting of H3 and H3K27me3. We observed upon quantification of the ratio of H3K27me3 to H3 in a set of biological triplicates that 2i mESCs treated with Chiron-124 lost 39% global H3K27me3 modification, trending towards significance (S8 Fig). These data support our model that G1 length significantly influences H3K27me3 levels, in both differentiated and pluripotent cell contexts.

To profile locus-specific changes due to Chiron-124, we performed H3K27me3 CUT&Tag after 48 h of treatment. We defined genome-wide H3K27me3 domains and generated unique segments from domain comparison between the treatment and control samples. To visualize H3K27me3 changes within these unique segments, we plotted the distribution of the log_2_ ratio of H3K27me3 levels at these unique segments in Chiron-124 versus DMSO-treated cells (Fig 5E). This distribution was left shifted relative to zero, indicating a global loss in H3K27me3, reflecting results obtained by immunoblot. We fit a bimodal distribution and defined three clusters based on their specific H3K27me3 change after G1 shortening – regions with lower H3K27me3 in Chiron-124 (CL1), regions with equivalent H3K27me3 between Chiron-124 and DMSO (CL2), and regions with higher H3K27me3 in Chiron-124 (CL3). H3K27me3 domains defined in DMSO-treated cells that intersected with CL1 segments featured a loss in H3K27me3 across their length upon Chiron-124 treatment and also appeared to shrink from their boundaries (Fig 5F). Domains that intersected with CL3 segments, much fewer than CL1, featured a gain in H3K27me3 (Fig 5F).

Next, we categorized segments within the three clusters based on their pattern of H3K27me3 change in short G1 phase cells compared to asynchronous (Fig 5G). CL1 and CL2, which cover far more base pairs than CL3, comprise segments that have shrunk compared to their corresponding domains in DMSO-treated cells. Thus, the global loss in H3K27me3 is manifested as the shrinking of individual domains on the genome, pointing to less time for PRC2 to spread the modification under a short G1 phase. In summary, in differentiated cells, shortening G1 results in an overall loss of H3K27me3, showing that G1 length influencing global H3K27me3 levels might be applicable across cell types.

### Weakening of the G1 checkpoint correlates with smaller H3K27me3 domains

Inactivation of retinoblastoma protein (Rb) and cyclin-dependent kinase inhibitor 2A (CDKN2A) occurs frequently in cancer [20,21]. Inactivation of these proteins is expected to weaken the G1 checkpoint, and we hypothesized that these perturbations would affect H3K27me3 domains due to reducing G1 length. To test this hypothesis, we defined H3K27me3 domains using SEGWAY [22] data from ENCODE [23] for cell lines that had inactivated Rb (HeLa, WERI-Rb-1) or had a CDKN2A deletion (A673, Panc1, SJSA1, and MCF-7), or neither (A549, HepG2, K562, SK-N-SH, Karpas-422, MM.1S, and PC-3). We coalesced segments defined as “FacultativeHet” by SEGWAY whose ends were within 500 bp of similar segments and then plotted the size distribution of H3K27me3 domains across the three categories. We observed the loss of CDKN2A and Rb to result in significantly smaller domains, with Rb loss having a bigger effect than CDKN2A (Fig 5H). Thus, complementary to the abrogation of the G1 checkpoint using Chiron-124 treatment, genetic weakening of the G1 checkpoint also correlates with smaller H3K27me3 domains, possibly due to a shorter G1 phase in these cells.

### Recovery of H3K27me3 in DMG cells with the H3K27M mutation

Our results in mESCs and HEK293 cells point to the importance of the length of the G1 phase in maintaining low H3K27me3 levels genome-wide. This modulation of heterochromatin may provide therapeutic relief in diseases where aberrant heterochromatin is associated with pathogenicity. One such disease is diffuse midline glioma (DMG), where children present with CNS tumors bearing an H3K27M mutation in one of the multiple copies of H3, resulting in a dominant negative effect of a global loss in H3K27me3 [24]. The substoichiometric H3K27M can lead to a spreading defect in PRC2 [25]. If H3K27me3 levels are substantially lowered in DMG cells due to reduced PRC2 activity, increasing cell cycle time could rescue H3K27me3 levels by giving PRC2 more time to modify H3 even at reduced efficiency. The CDK4/6 inhibitor Palbociclib restricts cells in the G1 phase via inhibition of the CDK4/6-cyclin D1-Rb pathway [26,27]. As DMG cells often exhibit elevated levels of CDK4 and CDK6, previous studies have investigated Palbociclib as a potential therapeutic option for patients [28,29]. Application of Palbociclib in vitro leads to inhibition of cell proliferation, and in vivo to the prolonged survival of mice implanted with DMG cells [27,28]. Some attention has been given to transcriptomic changes following Palbociclib treatment [29]; however, there has been comparatively little investigation into the drug’s impact on the epigenetic landscape characteristic of H3K27M-possessing DMG cells.

**Fig 6:**
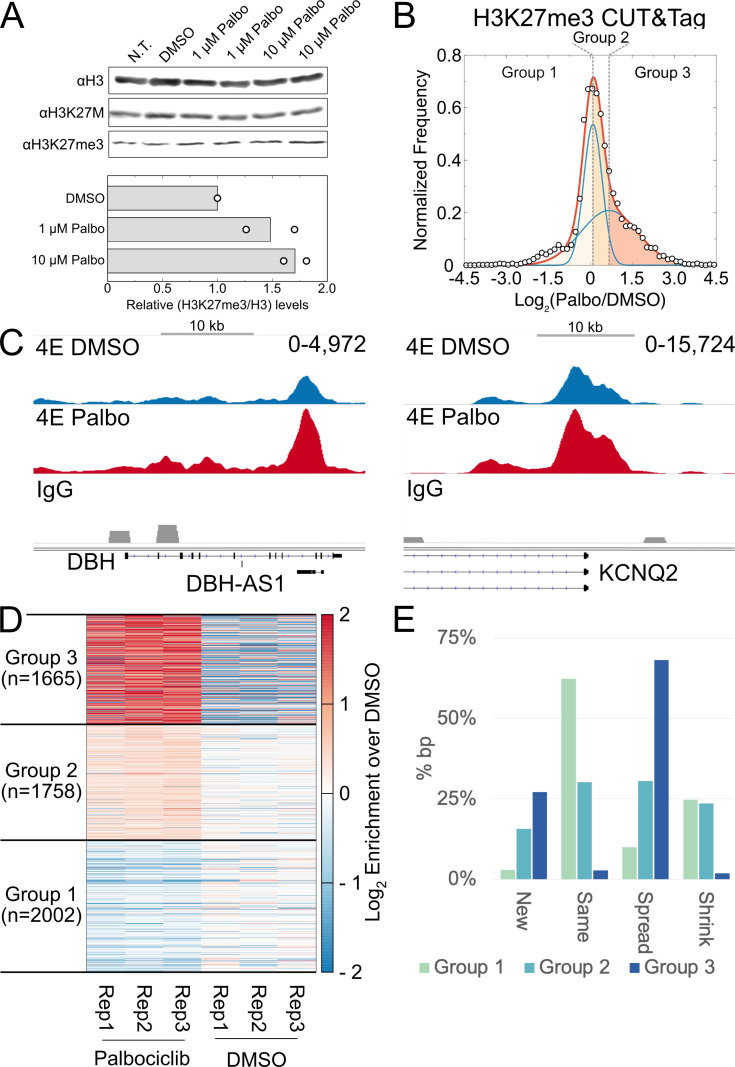
H3K27me3 domains are restored upon G1 arrest in a patient-derived H3.1K27M mutant DMG cell line. (A). Immunoblot for H3, H3K27M, and H3K27me3 after 72-h treatment with Palbociclib (top). Quantification of the modification levels normalized to DMSO-treatment (bottom). (B). Plot of log_2_ ratio of H3K27me3 levels in Palbociclib compared to DMSO-treated SU-DIPG-IV cells for unique domain segments. The H3K27me3 levels were determined from combining three replicates for each condition. The distribution was fitted with the sum of two normal distributions (plotted in red). The individual distributions are plotted in blue. The shaded regions correspond to the three groups based on the log_2_ ratio: loss of H3K27me3 upon treatment with Palbociclib (CL1, leftmost region), equivalent H3K27me3 in Palbociclib and DMSO treatment (CL2, center region), and regions with more H3K27me3 in cells treated with Palbociclib (CL3, rightmost region). (C). Representative H3K27me3 domains from the H3K27me3 gain CL3. (D). Heatmap of H3K27me3 CUT&Tag enrichment at individual replicates for SU-DIPG-IV cells treated with Palbociclib compared to DMSO vehicle control for the 3 clusters defined in (B). (E). Percentage of bp covered by segments belonging to each of the four categories of domain changes (shrinking, same, spreading, and new) observed in the 3 clusters defined in (B). Most domains present in CL1 after Palbociclib treatment maintain the same boundaries as observed in DMSO vehicle control samples. CL3 however, which presents the highest log_2_ enrichment of H3K27me3 after Palbociclib treatment, is mostly composed of domains with spreading boundaries that extend past those observed in DMSO control samples. The data underlying Fig 6A, 6B, 6D, 6E can be found in S6 Data, and the data underlying Fig 6C available at accession GSE264215 in GEO.

We asked if treatment with Palbociclib, by extending G1, would result in increased levels of H3K27me3 in the DMG cell line SU-DIPG-IV, which carries the H3.1K27M mutation. H3K27me3 levels but not H3.1K27M levels increased with 72-h Palbociclib treatment (Fig 6A). To investigate changes in H3K27me3 distribution after treatment with Palbociclib, we performed CUT&Tag in triplicate on H3K27me3 after 72-h Palbociclib treatment. As previously described, domains were defined for control and Palbociclib and then compared with each other to identify unique segments. The distribution of the log_2_ ratio of H3K27me3 enrichment between Palbociclib and DMSO-treated cells showed a clear bimodal distribution that could be fitted with two normal distributions (Fig 6B). The mean of the left-shifted distribution was approximately 0, whereas the mean of the right-shifted distribution was approximately 0.7. This allowed us to separate the segments into three groups based on the means of the two underlying distributions: regions that have a minor loss in H3K27me3 (Group 1), regions that have a minor gain in H3K27me3 (Group 2), and regions that have a substantial gain H3K27me3 (Group 3) after Palbociclib treatment (Fig 6B and 6D). Representative regions with H3K27me3 gain from Group 3 show a notable increase within known Polycomb domains present in the SU-DIPG-IV cells in combination with H3K27me3 extending beyond the established domain borders (Fig 6C). Indeed, the majority of the unique segments within Group 3 correspond to spreading from domain boundaries defined in the control treatment (Fig 6E). The observed overall increase in H3K27me3 across the SU-DIPG-IV cell line is notable, but it is unclear from this data alone if regions that gain H3K27me3 had lost it originally due to the H3K27M mutation present in the cells.

**Fig 7:**
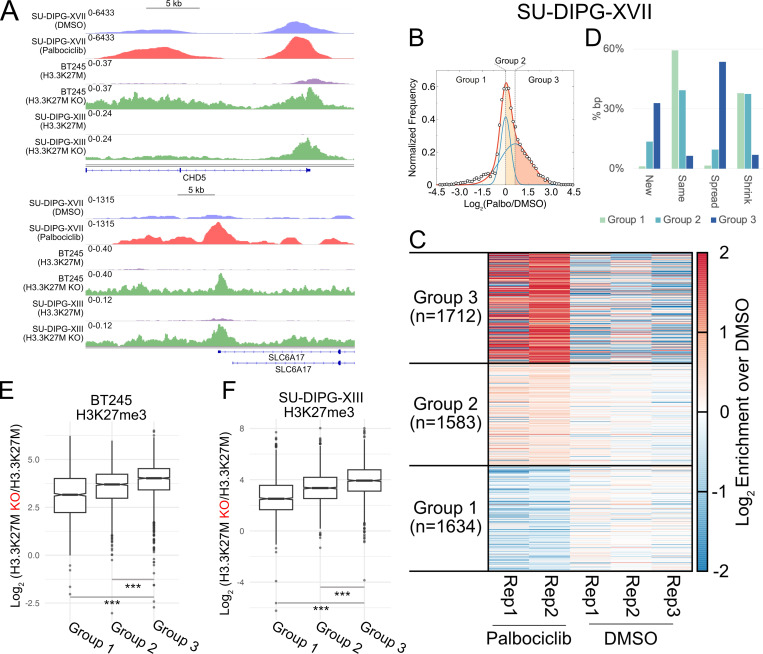
Restored H3K27me3 domains upon G1 arrest in a patient-derived H3.3K27M mutant DMG cell line mirror gains observed upon mutation deletion. (A). Representative H3K27me3 domains with gains in H3K27me3 upon G1 extension via Palbociclib treatment or knockout of H3.3K27M. (B). Plot of log_2_ ratio of H3K27me3 levels in Palbociclib versus DMSO-treated SU-DIPG-XVII cells for unique domain segments. H3K27me3 levels were calculated by combining two replicates of Palbociclib treatment and three replicates of DMSO treatment, respectively. Data points are shown as black outlined circles and are fitted with a bimodal distribution shown in red. This distribution is further separated into two normal distributions, plotted in blue. The three shaded regions represent groups with shared changes in H3K27me3: regions that lose H3K27me3 upon Palbociclib treatment (CL1, leftmost region), regions that minimally gain or maintain H3K27me3 upon Palbociclib treatment (CL2, center region), and regions that gain H3K27me3 upon Palbociclib treatment (CL3, rightmost region). (C). Heatmap of H3K27me3 CUT&Tag enrichment for individual replicates of SU-DIPG-XVII cells treated with Palbociclib compared to DMSO vehicle control for clusters defined in (B). (D). Percentage of bp covered by segments belonging to each of the four categories of domain changes (shrinking, same, spreading, and new) observed in the 3 clusters defined in (B). CL1 is composed primarily of maintained H3K27me3 domains between Palbociclib and DMSO treatments, along with some domains that present with shrinking boundaries. CL2 shows a similar trend of same and shrinking domains, with the addition of a small percentage of spreading domains. CL3 in comparison is composed predominantly of spreading domains that push past boundaries present in DMSO-treated cells, pointing to much of the observed H3K27me3 gains in this cluster arising from spreading of the mark. (E and F). Plots of log_2_ ratio of H3K27me3 levels in WT BT245 cells versus H3.3K27M KO cells **(E)** and in WT SU-DIPG-XIII cells versus H3.3K27M KO cells **(F)** for unique domain segments defined from Palbociclib treatment of SU-DIPG-XVII. CL3 shows the most gain of H3K27me3 in both KO BT245 cells and KO SU-DIPG-XIII cells, mirroring the gain that is seen in Palbociclib-treated SU-DIPG-XVII cells. The data underlying Fig 7B–F can be found in S7 Data, with data underlying Fig 7A available at accession GSE264215 in GEO.

We next asked if similar effects are observed in DMG cells carrying the H3.3K27M mutation. We performed H3K27me3 CUT&Tag after 72-h Palbociclib treatment in SU-DIPG-XVII cells, which carry the H3.3K27M mutation. As previously observed in SU-DIPG-IV cells, treatment with Palbociclib leads to a gain of H3K27me3 (Fig 7A). We proceeded to define genome-wide domains and generate unique segments that gain H3K27me3 in our CUT&Tag data upon Palbociclib treatment – independent from SU-DIPG-IV analysis. Plotting the log_2_ ratio distribution of Palbociclib-treated versus DMSO-treated SU-DIPG-XVII cells demonstrates a bimodal distribution fitted with two normal distributions that can be divided into three groups as seen in the H3.1K27M model: regions with H3K27me3 loss (Group 1), regions with limited H3K27me3 gain (Group 2), and regions with strong gain of H3K27me3 (Group 3) (Fig 7B and 7C). In defining the changes observed for each of these groupings, we observe a similar trend as was previously seen in Palbociclib-treated SU-DIPG-IV cells; namely, Group 3’s gain in H3K27me3 can be largely attributed to spreading of the modification past domain boundaries seen in DMSO-treated cells, with some contributions from new domain formation (Fig 7D). We next sought to compare regions with substantial gain in H3K27me3 upon G1 arrest in SU-DIPG-XVII to those observed in H3.3K27M knockout cells. We compared H3K27me3 ChIP-seq data from BT245 and SU-DIPG-XIII cell lines as reported by Harutyunyan and colleagues [25] to our CUT&Tag data from Palbociclib-treated SU-DIPG-XVII cells. In comparing domains present in Group 3, there is mirroring of H3K27me3 gains between the Palbociclib-treated cells and the two H3.3K27M knockouts in several regions of the genome (Fig 7A). To ask if this trend extended across the genome, we plotted the log_2_ ratio of H3K27me3 enrichment in the H3.3K27M KO cells versus cells containing H3.3K27M at segments from the three groups defined in our Palbociclib dataset. We observed a significantly higher gain of H3K27me3 due to H3.3K27M knockout at segments from Group 3 compared to Groups 2 and 1 (Fig 7E and 7F). The highest gain in H3K27me3 upon H3.3K27M deletion in the regions that also gain H3K27me3 during G1 arrest support our conclusion that modulation of the G1 phase length impacts the H3K27me3 landscape. These results further suggest that a longer G1 push H3.3K27M DMG cells toward a normal H3K27me3 profile.

Though the direct contribution of increased H3K27me3 spreading to cytostatic effects of Palbociclib in DMG cells is unknown, it is intriguing to note that extending G1 in these cells leads to increased H3K27me3 levels. As DMGs are defined by the co-occurrence of the H3K27M oncohistone and pathologically low levels of H3K27me3, G1 modulation may be a promising epigenetic therapy in this and other PRC2-deranged malignancies.

## Discussion

Here, we have demonstrated the interplay between H3K27me3 and the G1 length in human and mouse cells and shown that the length of the G1 phase influences the distribution and enrichment of H3K27me3 genome-wide. Upon manipulating the G1 phase, we observe domain-level changes of H3K27me3 spreading, shrinking, and de novo nucleation. Our results suggest that the G1 length plays a major role in shaping the epigenomic landscape, as observed in theoretical models [30].

In mESCs, G1 lengthening leads to global gain in H3K27me3 and domain-specific changes. These manipulations to the G1 phase were achieved in two capacities: an acute treatment of mESCs grown in serum with thymidine to measure immediate effects of G1/S arrest on H3K27me3 distribution, and a stable modulation of G1 length with mESCs grown in 2i medium for comparisons to the former condition. Serum-grown mESCs have hyperphosphorylated Rb and lack a G1 checkpoint, making this phase notably short, whereas 2i mESCs have an intact G1 checkpoint, leading to a significantly longer G1 than serum mESCs [9]. Beyond this change in cell cycle, several epigenetic differences have been reported between serum-grown and 2i mESCs. There is a global increase in the amount of H3K27me3 and a global decrease in CpG methylation in 2i compared to serum [13,31]. Others have observed that knockout of DNA methylation machinery in serum-grown mESCs results in spreading of H3K27me3 to regions with high CpG methylation in wild-type cells [32,33]. Indeed, enrichment of CpG dinucleotides is prevalent in canonical Polycomb targets and DNA methylation antagonizes PRC2 binding and H3K27me3 deposition [32,33]. In contrast to serum mESCs, loss of DNA methylation does not correlate with loss of H3K27me3 at the majority of Polycomb domains in 2i mESCs [34]. While DNA hypomethylation is likely an important factor contributing to remodeling of the H3K27me3 landscape within 2i mESCs compared to serum-grown cells, our results in thymidine-treated serum mESCs indicate that the observed de novo H3K27me3 domains are largely not within CpG-enriched regions of the genome (Fig 2D). The domains gained upon G1 extension in serum mESCs that mirror those gained in 2i mESCs may therefore point to a subset of Polycomb domains that are shaped in part by the longer G1 phase of these cells. Our results point to the possibility that changes in cell cycle length through development could provide a potential mechanism for rapidly changing the H3K27me3 landscape in addition to other known epigenetic changes like DNA methylation.

Rapid cell cycles in early development are a common theme in metazoans, making our observed influence of this cell cycle speed on the H3K27me3 landscape widely applicable. In *Drosophila* embryos, the establishment of the repressive chromatin modification H3K9me3 is influenced by changes to interphase duration that allow for cumulative recruitment of the methyltransferase Eggless [35]. Thus, constitutive and facultative heterochromatin may have a similar dependence on cell cycle timing for establishment during early development. While these results support our model of G1 length as a regulator of H3K27 methylation states in mESCs, there are still unanswered questions about how histone modifications other than H3K27me3 respond to G1 phase extension. Our MS data do suggest an increase in H3K36 and H3K9 methylation with longer G1. Determining whether competition may exist between histone-modifying enzymes due to changes to the cell cycle would be an interesting future avenue of research.

We observed the enrichment of H3K27me3 at new domains that do not overlap with known PRC2 recruitment features, while there is a notable loss of H3K27me3 enrichment within CpG islands and bivalent domains. Sequence and protein interactions may aid PRC2 recruitment to the newer, weaker nucleation sites exposed by longer G1. Similarly, changes in local methyltransferase and demethylase activity could help restructure the epigenome as the stem cell changes its cell cycle in preparation for differentiation.

In support of G1 length as a major determinant of global H3K27me3 levels, forcing HEK293 cells to proceed faster through the G1 phase causes a loss of H3K27me3. Our results agree with the previous finding that most global H3K27me3 recovery occurs in G1 in differentiated cells [6]. An acceleration in the cell cycle also occurs during T-cell activation. Upon exposure to an antigen, CD8^+^ T cells undergo rapid proliferation controlled by the phosphorylation state of Rb and, therefore, the duration of the G1 phase [36]. However, it is unknown how the heterochromatin landscape responds to these sudden rapid cell cycles. Though the cells up-regulate Ezh2 levels during activation [37] presumably to keep up with faster cell cycles, it is not known if this increased PRC2 activity during short cell cycles would be uniform across the genome. Upregulation of Ezh2 accompanies cell cycle shortening during B cell development as well [38]. The fact that activating mutations in Ezh2 leads to follicular and diffuse B cell lymphoma at a stage where the B cell cell cycle shortens points to the possibility that cell cycle shortening during the activation of immune cells could lead to heterochromatin remodeling [39].

While G1 length is notably different between cell types, as exemplified in T- and B-cells, activity of Polycomb complexes could also impact the H3K27me3 landscape. The expression of PRC2 subunits can vary within different cellular contexts [3], with a decrease in PRC2 levels observed during differentiation [12]. Quantitative mass spectrometry has shown that 2i mESCs possess higher levels of the core PRC2 subunits EZH2 and SUZ12 relative to serum mESCs [13]. However, our results show modulation of H3K27me3 levels globally and at specific genomic regions as G1 length is altered in several cell contexts. Both the differentiated HEK293 cells and pluripotent 2i mESCs lose H3K27me3 upon shortening G1, within the span of several normal divisions despite their different PRC2 abundances and compositions. G1 length is likely not the sole contributor to shaping the H3K27me3 landscape in cells but demonstrates a substantial impact on the epigenome, providing an additional layer of regulation to heterochromatin structure maintenance.

The chemical inhibition methods described in this work are effective for transient control of the G1 phase in both pluripotent and differentiated cells but may cause DNA damage that in turn could influence H3K27me3 levels. Previous studies have reported the localization of PRC2 at sites of DNA damage [40,41]. However, there is evidence pointing to H3K27me3 existing transiently at such sites [40], along with negligible colocalization of PRC2 and H3K27me3 with DNA damage sites in undifferentiated mESCs [42]. Undifferentiated mESCs are also far more tolerant of DNA damage [43]. Additionally, the correlation we see between domains upon thymidine treatment versus 2i medium further supports the model that our observations reflect recruitment and spreading of PRC2 that is not a significant part of a DNA damage response. Finally, we do not find GO terms related to DNA damage enriched in genes upregulated due to 20-h thymidine block from our RNA-seq dataset. For inhibition of Chk1 in differentiated cells, we do find the possibility of micronuclei in our flow cytometry data, pointing to DNA damage with the Chiron-124 treatment (Fig 5B). Most of the literature points to H3K27me3 gains at DNA damage sites, but we observe a strong global loss of H3K27me3. There are some minimal gains of trimethylation in CL3 in this set of experiments that may be influenced by DNA damage, but the overall effect of Chk1 inhibition on the H3K27me3 landscape points to mechanisms other than DNA damage influencing its shrinkage.

Finally, we observed increased H3K27me3 levels in DMG cells with the dominant negative H3K27M mutation when treated with the selective CDK4/6 inhibitor Palbociclib. Nucleosomes containing mutant H3K27M can directly interact with PRC2 to prevent PRC2 spreading [44]. Previous work in the DMG context has shown the importance of PRC2 to H3K27M ratio in eliciting the characteristic loss of H3K27me3; the larger the excess of H3K27M, the more robust inhibition of H3K27me3 spreading [12]. This ratio becomes even more unbalanced via PRC2 levels further decreasing as mutant mESCs differentiate to neuronal cell types, subsequently losing more H3K27me3. Our results support H3K27M cells displaying a PRC2 spreading defect that can be partially compensated by lengthened G1, without intervening to balance the PRC2 to H3K27M ratio. Our results would also suggest a loss of H3K27me3 in cancers that lose the G1 checkpoint via mutations in Rb or deletions in Cdkn2a. In these tumors, optimal PRC2 function might be critical to maintaining H3K27me3 under fast cell cycles similar to serum mESCs, pointing to PRC2 as a potential target in these tumors. In summary, the cell cycle could control H3K27me3 dynamics in disparate systems, such as early development and tumor growth.

## Methods

### Mammalian cell culture

All cell lines used in this work were tested for mycoplasma and confirmed to be negative for each experiment. Experiments utilized mouse embryonic stem cell line E3 (mESCs; Gates Biomanufacturing Facility at University of Colorado Denver), human embryonic kidney 293 cell line (HEK293; Fred Hutchinson), and patient-derived K27M SU-DIPG-IV (IV, H3.1K27M; J. Olson and J. Sarthy laboratories).

mESCs were cultured under feeder-free conditions on 0.1% gelatin-coated T-25 flasks in knockout DMEM (Life Technologies), supplemented with 20% embryonic stem cell-qualified fetal bovine serum (FBS; Life Technologies), 2 mM L-glutamine (Life Technologies), 0.1 mM non-essential amino acids (Life Technologies), 100 U mL^–1^ penicillin/streptomycin (Life Technologies), 0.05 mM β-mercaptoethanol (Sigma), and 1,000 U mL^–1^ ESGRO mouse leukemia inhibitory factor medium supplement (LIF; Millipore).

For the comparison of serum/LIF and 2i conditions, Mouse embryonic stem cell lines (mESCs) were cultured on gelatin-coated plates at 37 °C with 5% CO_2_ under either standard Serum/LIF conditions (KO-DMEM, 2 mM Glutamax, 15% ES grade fetal bovine serum, 0.1 mM 2-mercaptoethanol, 1× Pen/Strep, 1× NEAA and leukemia inhibitory factor (LIF)) or in 2i media (1:1 mix of DMEM/F12 and Neurobasal medium including N-2 supplement, B-27 supplement, 0.1 mM 2-mercaptoethanol, 2 mM glutamax, LIF, 3 µM CHIR99021 and 1 µM PD0325901).

HEK293 cells were cultured in DMEM (Life Technologies), supplemented with 10% FBS (Life Technologies) and 100 U/mL penicillin/streptomycin (Life Technologies). Cell lines were grown at 37 °C with 5% CO_2_ in a humidified Forma Steri-Cycle CO_2_ incubator (ThermoFisher Scientific) and subcultured approximately every 2 days to maintain exponential growth. To harvest cells, a 0.25% trypsin-EDTA solution (Gibco) was applied for 3–4 min for mESCs and 1 min for HEK293 cells. Detached cells were centrifuged at 2,000 rpm and 4 °C for 5 min and washed with 1× phosphate-buffered saline (PBS pH 7.4; Gibco) for use downstream.

The SU-DIPG-IV cell line had targeted sequencing to confirm H3 mutational status and was cultured as previously described [45].

### Thymidine treatment for G1/S arrest

For experiments utilizing thymidine (Sigma) to block cells in the G1/S phase, a final concentration of 2 mM thymidine was administered. The double-block experiments were performed by first treating mESCs with thymidine for 24 h, releasing them for 7 h via 1× PBS wash and recovery in a fresh medium, followed by a second block for 20 h. In preparation for CUT&RUN, mESCs were treated with thymidine for 8, 12, 16, and 20 h before being harvested together with asynchronous cells as control. These samples were then immediately processed for CUT&RUN.

### RO-3306 treatment for mESC cell cycle length determination

For experiments utilizing RO-3306 (MedChemExpress) to block cells in the G2 phase, a final concentration of 10 µM was administered. The block-and-release experiment was performed by treating mESCs with RO-3306 for 15 h, followed by a wash with 1× PBS and the addition of fresh medium to cells. Samples were harvested for flow cytometry as described below immediately after release and 2-, 4-, 6-, 8-, 10-, 12-, 14-, and 16-h post-release.

### Chiron-124 treatment for G1 shortening

For experiments utilizing Chiron-124 (MedChemExpress) to shorten the G1 phase for differentiated cells, a final concentration of 0.5 µM was administered to cells. To determine how G1 shortening affected global H3K27me3, HEK293 cells were treated with Chiron-124 for 48 h while 2i mESCs were treated with Chiron-124 for 20 h, then harvested for flow cytometry and histone acid extraction followed by immunoblot as described below. To determine domain-level H3K27me3 changes in HEK293 cells after G1 shortening, cells were treated with Chiron-124 for 48 h and then immediately processed for CUT&Tag.

### Palbociclib treatment for G1 arrest

For experiments utilizing Palbociclib (Cell Signaling Technologies) to arrest SU-DIPG-IV cells in the G1 phase, cells were treated for 72 h before harvesting CUT&Tag experiments. For CUT&Tag profiling, cells were treated with 10 µM Palbociclib before being harvested for immediate use.

### Flow cytometry for cell cycle gating

Cells were harvested using a trypsin solution as detailed above and washed with 1 mL 1× PBS before being pelleted at 2,000 rpm and 4 °C for 5 min. The cells were then resuspended in 70% ethanol via gentle vortexing and incubated overnight at −20 °C. Cells were pelleted at 2,000 rpm and 4 °C to remove ethanol for 5 min. Cells were resuspended in 500 µL Krishan stain [46] (9.25 mM sodium nitrate, 69 µM prodium iodide, 0.01% NP40, and 2 mg RNaseA) and incubated overnight at 4 °C. HEK293 samples were analyzed using a Beckman Coulter FC500 flow cytometer using 488 nm laser for PI. mESC samples for cell cycle timing quantification were run in a Beckman Coulter Gallios 561 flow cytometer using 488 nm laser for PI. Cell gating was performed using the FlowJo (BD Life Sciences) and RStudio softwares. Raw FCS files for flow cytometry experiments are available in the FlowRepository public repository under accessions FR-FCM-Z96X and FR-FCM-Z96P.

### Histone purification via acid extraction

Histones were purified from flash-frozen cells using a standard acid extraction protocol adapted from Shechter *and colleagues* [47]. Cells were thawed on ice and then lysed in hypotonic lysis buffer (10 mM Tris-HCl pH 8.0, 1 mM KCl, 1.5 mM MgCl_2_, and 1 mM DTT). Intact nuclei after lysis were pelleted by centrifugation at 10,000× *g* and 4 °C for 10 min before being resuspended in 0.4 N H_2_SO_4_ and incubated at 4 °C overnight. After incubation, nuclear debris after lysis was pelleted via centrifugation at 16,000× *g* and 4 °C for 10 min. Histones were precipitated via the dropwise addition of 150 µL 100% trichloroacetic acid and then incubated on ice for 30 min. The precipitated histones were pelleted via centrifugation at 16,000× *g* and 4 °C for 10 min, washed once with cold acetone, and then air-dried at room temperature for 20 min. The final histone pellet was then dissolved in 100 µL DI water. The concentration of histones in each sample was determined using the BCA protein assay kit (ThermoFisher Scientific).

### Immunoblotting analysis

Acid-extracted histones were prepared in 1 µg samples, separated by electrophoresis using a 15% SDS-polyacrylamide gel, and transferred to a nitrocellulose membrane at room temperature. Whole-cell extracts were separated using a 4–20% Tris-Glycine polyacrylamide gel (Invitrogen) and transferred to a nitrocellulose membrane. Histones isolated from mESCs and HEK293 cells treated with thymidine and Chiron-124, respectively, were probed with 1:1000 dilutions of antibodies against H3K27me3 (#9733, Cell Signaling Technologies) and total H3 (#18521, Abcam). Following overnight primary antibody incubation, membranes were probed with 1:10000 dilutions of IRDye 680RD Goat anti-Mouse IgG (926-68070, LICORbio) and IRDye 800CW Goat anti-Rabbit IgG (926-32211, LICORbio) and imaged with a Sapphire Biomolecular Imager (Azure Biosystems) using 784/BP22 and 658/BP22 membrane focus settings. Signals for each blot were quantified using the ImageJ software as follows [48]. Raw tiff images were loaded into the ImageJ software, split into separate images for each color channel used in imaging, and each was set to an 8-bit inverted image. The gel analysis tools were used to determine the signal for each band. The ratio of H3K27me3 to H3 signal was determined for each sample and normalized to the asynchronous ratio for plotting.

### Mass spectrometry analysis of H3K27me3

Aliquots corresponding to 10 µg of enriched histones in water were placed in Eppendorf tubes and taken to dryness by vacuum centrifugation. Histone derivatization and digestion were carried out similarly to that described by Sidoli *and colleagues* [49]. Dried samples were resuspended in 20 µL of 100 mM ammonium bicarbonate, followed by the sequential addition of 5 µL acetonitrile, 5 µL propionic anhydride, and 14 µL ammonium hydroxide. The derivatization reaction was incubated at 37 °C with mixing for 15 min and then dried by vacuum centrifugation. The derivatization reaction was repeated once and dried by vacuum centrifugation to approximately 10 µL. Enzymatic digestion was carried out by adding 50 µL of 1 M ammonium bicarbonate to neutralize the solution and 1 µg of trypsin, after which the digestion reaction was incubated overnight at 37 °C with mixing. Samples were dried to approximately 20 µL, and peptide derivatization was carried out twice, as previously detailed, ending with dried samples. The samples were resuspended in 20 µL of 0.1% trifluoroacetic acid, desalted with C18-micro ZipTips (Millipore), and taken to dryness. LC-ESI-MS/MS (liquid chromatography coupled to electrospray ionization tandem mass spectrometry) was carried out with an Orbitrap Fusion (ThermoFisher Scientific) mass spectrometer using an instrument configuration and data-independent acquisition (DIA) for bottom-up proteomics as described by Karch *and colleagues* [50]. DIA data were analyzed using EpiProfile v2.0 [51]. EpiProfile generated extracted ion chromatograms for each peptide group (a peptide with different modification isoforms) and calculated the area under the curve (AUD) for each isoform. The software calculated the relative abundance of each isoform by dividing the AUC of a given isoform by the summed total AUC for all isoforms for that peptide. Biological triplicates were analyzed, and average modification percentages and standard deviations are reported. Raw mass spectrometry files and EpiProfile parameters and ratio files are available in the MassIVE public repository under accession MSV000093658.

### CUT&RUN

Genome-wide H3K27me3 domain changes were determined using the CUT&RUN protocol adapted from Hainer and Fazzio [52]. Cells were harvested via 0.25% trypsin-EDTA solution and counted using 0.4% trypan blue stain and countess automated cell counter (Invitrogen). A single-cell suspension was then prepared to achieve 0.5 x 10^6^ cells in 1 mL cold nuclear extraction buffer (20 mM HEPES-KOH pH 7.9, 10 mM KCl, 0.5 mM spermidine, 0.1% Triton X-100, and 20% glycerol). Isolated nuclei were pelleted via centrifugation at 2,000 rpm and 4 °C for 5 min, then resuspended in 600 µL cold nuclear extraction buffer. Biomag Plus Concanavalin A beads (Bangs Laboratories) were activated via two washes with 1 mL cold binding buffer (20 mM HEPES-KOH pH 7.5, 10 mM KCl, 1 mM CaCl_2_, and 1 mM MnCl_2_) using a magnetic rack, with 150 µL bead slurry used for each sample of 0.5 × 10^6^ cells. After the final wash, beads were resuspended in 300 µL cold binding buffer. While gently vortexing, nuclei were added to activated beads and then incubated on a rotating platform at 4 °C for 10 min to facilitate nuclei binding. Nuclei:bead samples were resuspended in 1 mL blocking buffer (20 mM HEPES pH 7.5, 150 mM NaCl, 0.5 mM spermidine, 0.1% BSA, 2 mM EDTA, and protease inhibitors) and incubated at room temperature for 5 min. After blocking, samples were washed with 1 mL wash buffer (20 mM HEPES pH 7.5, 150 mM NaCl, 0.5 mM spermidine, 0.1% BSA, and protease inhibitors) then resuspended in 250 µL wash buffer. While gently vortexing, 5 µg of anti-H3K27me3 (#9733S, Cell Signaling Technology) in 250 µL wash buffer was added to each sample. Antibody binding was facilitated by incubating samples on a rotating platform at 4 °C overnight. Samples were washed twice with 1 mL cold wash buffer and then resuspended in 250 µL cold wash buffer. While gently vortexing, 2.5 µL of CUTANA pAG-MNase (EpiCypher) was added, and samples were incubated at 4 °C for 1 h to facilitate binding. After incubation, samples were washed twice with 1 mL cold wash buffer and resuspended in 50 µL cold wash buffer. While gently vortexing, 3 µL 0.1 M CaCl_2_ was added to each sample. Samples were briefly flicked to mix and then incubated on ice for 30 min after which the pAG-MNase was inactivated by adding 50 µL 2× STOP buffer (340 mM NaCl, 20 mM EDTA, 4 mM EGTA, and 0.05% digitonin) supplemented with RNase A (ThermoFisher Scientific), proteinase K (ThermoFisher Scientific), glycogen (VWR), and spike-in DNA generated from MNase digestion of *Drosophila* S2 cells. Released fragments were cleaned up by incubating samples at 37 °C for 20 min, followed by centrifugation at 16000*g* and 4 °C for 5 min. The resulting supernatant was mixed with 3 µL 10% SDS and 2.5 µL proteinase K solution, followed by incubation at 70 °C for 10 min. To purify MNase-digested DNA fragments, samples underwent phenol-chloroform extraction. The final resulting aqueous phase was transferred and incubated with 2 µg glycogen and 750 µL ethanol at −20 °C overnight. DNA was pelleted by centrifugation at 16000× *g*, air-dried, and then resuspended in 0.1× TE buffer. DNA fragments were checked for quality using the Qubit dsDNA HS Assay kit (Invitrogen) and TapeStation High Sensitivity DNA assay kit (Agilent).

Sequencing libraries of DNA fragments isolated following CUT&RUN were prepared using the NEBNext Ultra II DNA Library Prep Kit for Illumina (New England BioLabs) and the NEBNext Multiplex Oligos for Illumina (96 Unique Dual Index Primer Pairs; New England BioLabs). Libraries were checked for quality using the Qubit dsDNA HS Assay kit and TapeStation High Sensitivity DNA assay kit. Sequencing was performed using a NovaSeq 6000 with Illumina 150 bp paired-end reads at the Genomics Shared Resource, University of Colorado Cancer Center.

### RNA-seq

In preparation for RNA-seq, mESCs were treated in triplicate with thymidine at a final concentration of 2 mM for 20 h before being harvested together with asynchronous cells as control. Growth media was removed and 1 × 10^6^ cells were resuspended in 300 µL TRIzol (Invitrogen), homogenized, transferred to eppendorf tubes and frozen at –20 °C. Frozen samples were thawed and RNA extracted using the miRNeasy Micro Kit (Qiagen) with on-column DNAse I digestion. Extracted RNA was shipped to Novogene (California, USA), where RNA-seq libraries were generated. Messenger RNA was purified from total RNA using poly-T oligo-attached magnetic beads. After fragmentation, the first strand cDNA was synthesized using random hexamer primers followed by the second strand cDNA synthesis. The library was ready after end repair, A-tailing, adapter ligation, size selection, amplification, and purification. The library was checked with Qubit and real-time PCR for quantification and bioanalyzer for size distribution detection. Quantified libraries were pooled and sequenced on Illumina platforms.

### Chromatin immunoprecipitation (ChIP)

Native ChIP was performed with 2 × 10^6^ cells as previously described [53]. Cells were trypsinized, washed and subjected to hypotonic lysis (50 mM TrisHCl pH 7.4, 1 mM CaCl_2_, 0.2% Triton X-100, 10 mM NaButyrate, and protease inhibitor cocktail (Roche)) with micrococcal nuclease for 5 min at 37 °C to recover mono- to tri-nucleosomes. Nuclei were lysed by brief sonication and dialyzed into RIPA buffer (10 mM Tris pH 7.6, 1 mM EDTA, 0.1% SDS, 0.1% Na-Deoxycholate, 1% Triton X-100) for 2 h at 4 °C. Soluble material was incubated with 3 μg of antibody bound to 50 μl protein A Dynabeads (Invitrogen) and incubated overnight at 4 °C, with 5% reserved as input DNA. Magnetic beads were washed as follows: 3× RIPA buffer, 2× RIPA buffer + 300 mM NaCl, 2× LiCl buffer (250 mM LiCl, 0.5% NP-40, 0.5% NaDeoxycholate), 1× TE + 50 mM NaCl. Chromatin was eluted and treated with RNaseA and Proteinase K. ChIP DNA was purified and dissolved in H_2_O.

### ChIP-seq library preparation

ChIP-seq libraries were prepared from 5–10 ng ChIP DNA following the Illumina TruSeq protocol. The quality of the libraries was assessed using a D1000 ScreenTape on a 2200 TapeStation (Agilent) and quantified using a Qubit dsDNA HS Assay Kit (Thermo Fisher). Libraries with unique adaptor barcodes were multiplexed and sequenced on an Illumina NextSeq 500 (paired-end, 33 base pair read). Typical sequencing depth was at least 20 million reads per sample.

### CUT&Tag

Genome-wide H3K27me3 domain changes were profiled using the CUT&Tag protocol adapted from EpiCypher. Cells were harvested via 0.25% trypsin-EDTA solution and counted using 0.4% trypan blue stain and Countess automated cell counter. A single-cell suspension was prepared by washing cells with cold 1x PBS and resuspending 1.0 × 10^6^ cells in 1 mL cold nuclear extraction buffer (20 mM HEPES-KOH pH 7.9, 10 mM KCl, 0.1% Triton X-100, 20% glycerol, and 0.5 mM spermidine), then incubated on ice for 10 min to isolate nuclei. Biomag Plus Concanavalin A beads were activated with two washes of cold bead activation buffer (20 mM HEPES-KOH pH 7.9, 10 mM KCl, 1 mM CaCl_2_, and 1 mM MnCl_2_). After activation, beads were added to aliquots of 0.1 × 10^6^ isolated nuclei and incubated at room temperature for 10 min. Nuclei:bead samples were resuspended in 50 µL cold Antibody150 buffer (20 mM HEPES-KOH pH 7.5, 150 mM NaCl, 0.5 mM spermidine, 0.01% digitonin, 2 mM EDTA, and protease inhibitors) followed by the addition of 0.5 µg of anti-H3K27me3 (#9733S, Cell Signaling Technology). To facilitate primary antibody binding, samples were incubated on nutator at room temperature for 1 h. Samples were resuspended in 50 µL Digitonin 150 buffer (20 mM HEPES-KOH pH 7.5, 150 mM NaCl, 0.5 mM spermidine, 0.01% digitonin, and protease inhibitors) followed by the addition of 0.5 µg CUTANA anti-rabbit secondary antibody (#13-1047, EpiCypher). Samples were then incubated on nutator at room temperature for 1 h to facilitate binding, followed by two washes with 200 µL cold Digitonin 300 buffer (20 mM HEPES-KOH pH 7.5, 300 mM NaCl, 0.5 mM spermidine, 0.01% digitonin, and protease inhibitors). The tagmentation reaction was performed by resuspending samples in 50 µL cold Tagmentation buffer (20 mM HEPES pH 7.5, 300 mM NaCl, 0.5 mM spermidine, 10 mM MgCl_2_, and protease inhibitors) and incubating at 37 °C in thermocycler for 1 h. Samples were washed with 50 µL TAPS buffer (10 mM TAPS pH 8.5 and 0.2 mM EDTA) and then resuspended in 5 µL SDS Release buffer (10 mM TAPS pH 8.5 and 0.1% SDS) to quench the tagmentation reaction and release adaptor-tagged DNA fragments into solution. After incubating samples at 58 °C in thermocycler for 1 h, 15 µL SDS Quench buffer (0.67% Triton X-100), 2 µL i5 universal primer (EpiCypher), 2 µL i7 barcoded primer (EpiCypher), and 25 µL NEBNExt High Fidelity 2x PCR Master Mix were added to each sample for library construction with the following PCR protocol: 5 min at 58 °C, 5 min at 72 °C, 45 s at 98 °C, 14 cycles of 15 s at 98 °C and 10 s at 60 °C, with a final extension for 1 min at 72 °C.

Libraries were checked for quality using the Qubit dsDNA HS Assay kit and TapeStation High Sensitivity DNA assay kit, and then sequencing was performed using Novaseq 6,000 (Novogene) to generate 150 bp paired-end reads.

### CUT&Tag in SU-DIPG-IV cells

Nuclei were prepared from SU-DIPG-IV cells, and up to 5 × 10^5^ cells were used per individual CUT&Tag reaction using CUT&Tag V3 (https://www.protocols.io/view/cut-amp-tag-direct-for-whole-cells-with-cutac-x54v9mkmzg3e/v3). pAG-Tn5 was purchased from Epicypher (Durham, North Carolina), and H3K27me3 antibody was purchased from Cell Signalling Technologies (#9733). Rabbit Isotype IgG control (ab172730) was purchased from Abcam. Antibodies were used at 1:100 dilution. Sequencing was performed on an Illumina NextSeq 500 using a P1/100 cycle kit with 50 bp paired-end reads, targeting 10 million reads per sample.

### RNA-seq analysis

A concatenated fasta file was created using (i) cDNA sequences of Mus musculus genes (GRCm38/mm10 genome version, release 102 from Ensembl), (ii) whole genome sequence of Mus musculus (mm10 genome version). A Salmon index was created using the concatenated fasta, setting the whole genome sequences as decoys. Transcript quantification was performed using Salmon v1.9 [54] with libType set as automatic. Differential gene expression analysis was performed using DeSeq2 (v1.30.1) [55] in R (v4.0.3). The expression matrix across genotypes was transformed using the “rlog” function in DESeq2 and the transformed matrix was used for plotting normalized read counts of specific genes (S5A Fig) performing principal component analysis (PCA) using the “prcomp” function in R and plotted using ggplot2 (S5B Fig). Gene set enrichment analysis was performed using WebGestalt [56].

### Spike-in normalized CUT&RUN analysis

CUT&RUN sequencing reads from triplicate experiments were trimmed using Cutadapt [57]: Illumina adapter sequences were removed and reads trimmed to 140 bp. Reads less than 35 bp were discarded. Trimmed reads were aligned to both the mm10 version of *Mus musculus* genome and dm6 version of the *Drosophila melanogaster* genome using bowtie2 [58]. On average, 2.6% of mapped reads mapped to the *Drosophila* genome. Even with spike-in normalization, when comparing cells blocked in G1 with asynchronous cells, we will underestimate H3K27me3 gain. This is due to the fact that DNA content of G1-blocked cells will always be lower than asynchronous cells.

Samtools [59] and bedtools [60] were used for processing aligned reads from sam to bed files. Coverage at 100 bp windows of the mouse mm10 reference genome was calculated as number of reads of given length (120–500 bp for CUT&RUN) that mapped at that window normalized by the factor N:


N=10,000/(Total\ number\ of\ reads\ that\ mapped\ to\ Drosophila\ genome)

Here 10,000 is an arbitrarily chosen number. The number of normalized reads were combined across the three replicates for each time point.

All scripts used for domain calling are deposited in Zenodo under the repository: https://doi.org/10.5281/zenodo.6783950. The normalized read counts were then smoothed with a running average spanning ±1,000 bp around each 100 bp bin using gen_ra_noIN_v2.pl. Domain calling was performed using call_domains_v4.1_hardCutoff.pl. The distribution of normalized read counts in 100 bp windows genome-wide was generated, and a “domain cutoff” was determined as the normalized read count that is greater than the normalized read count of *F*% of the windows. *F* was set based on background signal as 95. Domains were called by linking adjacent windows with a normalized read count ≥ domain cutoff. To accougent for short disruptions due to issues of mappability, jumps of up to 750 bp, where normalized read count ≥0.75*domain cutoff was allowed while linking windows. A log_2_ ratio of H3K27me3 enrichment over IgG enrichment was calculated for all the putative domains. Those domains with a log_2_ enrichment > 2 (4-fold enrichment over IgG) were used for all downstream analyses. For performing disjoin and reduce operations, the GenomicRanges package in R was used [61]. *K*-means clustering was performed in R and resulting heatmap plotted using gnuplot. Intersection of unique segments from each cluster with domains defined in Asynchronous data was performed using bedtools [60].

### ChIP-seq analysis

ChIP-seq reads were aligned to the mm10 version of *Mus musculus* genome using bowtie2. Samtools [59] and bedtools [60] were used for processing aligned reads from sam to bed files. Coverage at 100 bp windows genome-wide was calculated as the number of reads that mapped at that window, normalized by the factor N:


N=2800000000/(Total\ number\ of\ mapped\ reads)

2800000000 was a number chosen arbitrarily in the range of total mapped base pairs in M. musculus genome. Domain calling was performed similar to CUT&RUN datasets. The distribution of log_2_ fold changes in 2i versus serum/LIF was plotted using Xmgr (https://plasma-gate.weizmann.ac.il/Xmgr/) which allows non-linear curve fitting. Bimodal normal distribution was fitted using the following formula:


$$\text{y}=\text{A}0*\text{exp}(-1*(((\text{x}-\text{A}1)/\text{A}2{)}^{2}))\;+\text{ A}3*\text{exp}(-1*(((\text{x}-\text{A}4)/\text{A}5{)}^{2}))$$

Where A1 and A2 represent the mean values for the two normal distributions. Hypergeometric test for overlaps in segments between 2i/Serum/LIF and thymidine experiments was performed using phyper function in *R* and multiple testing correction was performed to adjust *p*-values.

### CUT&Tag analysis

CUT&Tag sequencing reads were trimmed using Cutadapt [57]: Illumina adapter sequences were removed and reads trimmed to 140 bp. Reads less than 35 bp were discarded. Trimmed reads were aligned to either the mm10 version of *Mus musculus* genome or the hg38 version of the human genome using bowtie2 [58]. Samtools [59] and bedtools [60] were used for processing aligned reads from sam to bed files. Duplicate reads were discarded for further analysis if the reads had the same start and end coordinates. Coverage at 100 bp windows genome-wide was calculated as the number of reads (120–1000 bp in length) that mapped at that window, normalized by the factor N:


N=2800000000/(Total\ number\ of\ mapped\ reads)

2800000000 was a number chosen arbitrarily in the range of total mapped base pairs in M. musculus/human genome. The normalized coverage in 100 bp windows was used for the plots in Figs 3 and S6, and for genome browser snapshots. Domain calling for HEK293 cells and SU-DIPG cells were performed identically to CUT&RUN datasets. The “domain cutoff” (the normalized read count that is greater than the normalized read count of F% of the windows), set based on background signal as 95 for all datasets except for SU-DIPG-IV datasets, where it was set as 75.

The distribution of log_2_ fold changes (Figs 5E, 6B, and 7B) was performed the same way as described for 2i/Serum/LIF datasets above. For Fig 5F, normalized read counts were plotted relative to domain centers by first making a matrix of normalized read counts and plotting the matrix using gnuplot. Log_2_ ratio was calculated by dividing each element of the matrix for Chiron-124 treatment by the corresponding element of the matrix for DMSO treatment, and setting all values outside domain boundaries to 0. The matrices were plotted as heatmaps using gnuplot. For Figs 6D and 7C, normalized read counts were averaged at each segment of Groups 1–3 for each individual replicate. For each segment, the average enrichment across replicates of DMSO treatment was calculated and the log_2_ ratio of values for the segment across all replicates to the mean DMSO value was calculated. The resultant normalized matrix was plotted using gnuplot.

### ENCODE data analysis

For defining H3K27me3 domains from ENCODE, the following SEGWAY bed files were used. Control group: ENCFF489OJS.bigBed (A549), ENCFF248VPB.bigBed (HepG2), ENCFF097QCC.bigBed (K562), ENCFF412RIG.bigBed (SK-N-SH), ENCFF311LMS.bigBed (Karpas-422), ENCFF078XFK.bigBed (MM.1S), and ENCFF437JRM.bigBed (PC-3); Rb group: ENCFF835IDW.bigBed (WERI-Rb-1) and ENCFF137OLX.bigBed (HeLa-S3); CDKN2A group: ENCFF512MOL.bigBed (A673), ENCFF253DDW.bigBed (Panc1), ENCFF427CTV.bigBed (SJSA1), and ENCFF830MUH.bigBed (MCF-7).

### Analysis of H3K27me3 ChIP-seq data from Harutyunyan and colleagues [25]

Bam files of single-end H3K27me3 spike-in ChIP-seq data from BT245 and SU-DIPG-XIII cell lines (both H3.3K27M and H3.3K27M KO cell lines) were downloaded from links provided in the published paper. The original bam files were generated by aligning reads to hg19. Hence, we converted the bam files back to fastq files using the bedtools command “bamtofastq”. The single-end reads were then mapped independently to hg38 version of the human genome and dm3 version of *Drosophila* genome using bowtie2 [58]. SAMtools [59] and bedtools [60] were used for processing aligned reads from sam to bed files. Each read was then extended 250 bp (based on fragment lengths reported in the original paper) and coverage at 100 bp windows of the human hg38 reference genome was calculated as number of reads that mapped at that window normalized by the factor N:


N=10,000/(Total\ number\ of\ reads\ that\ mapped\ to\ Drosophila\ genome)

Here 10,000 is an arbitrarily chosen number. The number of normalized reads were combined across the two replicates for BT245 datasets. Then, average of the normalized read counts was then calculated for H3.3K27M and H3.3K27M KO datasets for each cell line at segments belonging to Groups 1–3 defined in Fig 7B and 7C. Then log_2_ ratio of the average enrichment at each segment for the H3.3K27M KO dataset to the average enrichment at the same segment for the H3.3K27M dataset was calculated separately for BT245 and SU-DIPG-XIII. Boxplots representing the log_2_ ratios for segments belonging to each group was generated using ggplot2 in R.

## Supporting information

S1 Raw Images PDF containing raw immunoblot images. (PDF)

S1 Fig Determination of serum/LIF mESC cell cycle length via G2/M block and release. **(A)**. Flow cytometry analysis of DNA content using propidium iodide fluorescence for asynchronous mESCs grown in serum/LIF medium. **(B–J)**. Same as (**A**) serum/LIF-grown mESCs treated with the Cdk1 inhibitor RO-3306 for 15 h followed by release from block for 0 h (**B**), 2 h (**C**), 4 h (**D**), 6 h (**E**), 8 h (**F**), 10 h (**G**), 12 h (**H**), 14 h (**I**), and 16 h (**J**). **(K)**. Quantification of percentage of cell population in each phase of the cell cycle (G1, S, and G2) for each release time point profiled in (**B**) through (**J**). Analysis shows that upon release from G2 arrest, mESCs take approximately 2–4 h to proceed through the G1 phase and a total cell cycle time of approximately 10 h (compare (**B**) and (**G**)). (**L**), (**M**) show the successive gates applied to the flow data before the analysis of PI histogram. Data underlying this figure can be found in S11 Data.
(PDF)

S2 Fig H3K27me3 is gained proportional to length of G1 arrest. Example tracks of normalized H3K27me3 CUT&RUN data at select genomic loci in the mouse genome after G1 arrest for 8, 12, 16, and 20 h via thymidine treatment. **(A–C)**. H3K27me3 enrichment at Gm36649 (**A**), Cdh23 (**B**), and Cpa1 (**C**). These genes present with H3K27me3 domains in asynchronous cells that shows gain both within the existing domain boundaries and spreading past these boundaries, the strength of which is proportional to the length of G1 arrest. **(D)**. H3K27me3 enrichment at region around Pla2g2f. Asynchronous mESCs show little enrichment while progressive G1 arrest leads to the establishment of a stronger H3K27me3 domain. The genomic snapshots were created using IGV, setting the midpoint of the data range as the lower cut-off used in calling domains. Thus, data above midpoint (red) would belong to domains, whereas data below midpoint (blue) would be outside domains.
(PDF)

S3 Fig Gene ontology analysis for regions with H3K27me3 loss upon G1 arrest. Gene ontology (GO) overrepresentation analysis was performed for genes belonging to clusters 1 and 2, defined from H3K27me3 CUT&RUN data in thymidine-treated serum/LIF-grown mESCs in Fig 2. Unique segments within both clusters lose H3K27me3 enrichment proportional to the length of G1 arrest, with cluster 1 presenting with more loss than cluster 2. GO analysis for both clusters reveals that genes contained in each are enriched for terms related to differentiation and development including cell fate commitment, pattern specification process, and embryonic organ development.
(PDF)

S4 Fig Gene ontology analysis for regions with H3K27me3 gain upon G1 arrest. Gene ontology (GO) overrepresentation analysis was performed for genes belonging to clusters 5 and 6, defined from H3K27me3 CUT&RUN data in thymidine-treated serum/LIF-grown mESCs in Fig 2. Unique segments within both clusters gain H3K27me3 enrichment proportional to the length of G1 arrest, with a majority of this gain observed outside of existing H3K27me3 domains in asynchronous cells with cluster 6 presenting with a stronger gain of H3K27me3 and greater proportion of new domains compared to cluster 5. GO analysis for cluster 5 reveals that genes contained in this cluster are enriched for terms related to immune function including adaptive immune response and cytokine receptor binding. GO analysis for cluster 6 reveals that genes contained in this cluster are enriched for terms related to the cytoskeleton and protease activity.
(PDF)

S5 Fig G1 arrest via thymidine treatment in mESCs shows reproducibility while maintaining pluripotency. **(A)**. Loadings of the first two principal components from principal component analysis of the three RNA-seq replicates after 20-h G1 arrest via thymidine treatment, along with asynchronous controls. Thymidine-treated samples separate from asynchronous controls while clustering together amongst themselves. **(B)**. Log_2_ normalized RNA counts of key pluripotency and differentiation markers from the RNA-seq replicates shown in (**A**). Expression of the pluripotency markers *Nanog* and *Oct4* are high in both thymidine-treated and asynchronous samples, while differentiation markers remain low in both conditions. Data underlying this figure can be found in S8 Data.
(PDF)

S6 Fig Correlation between H3K27me3 enrichment changes captured by CUT&RUN versus CUT&Tag. Plot of log_2_ ratio of H3K27me3 levels in thymidine-treated compared to asynchronous serum-grown mESCs for unique domain segments as measured by CUT&Tag (*x*-axis) versus CUT&RUN (*y*-axis) experiments. Hexagonal binning was performed using geom_hex in ggplot2 and a linear model fit is shown as a red line. Pearson correlation coefficient and the corresponding *p*-value for the comparison between the two datasets is shown on the top left corner of the plot. Data underlying this figure can be found in S9 Data.
(PDF)

S7 Fig H3K27me3-enriched domains in 2i mESCs reflect gains seen upon G1 arrest in serum-grown cells. Example tracks of normalized H3K27me3 ChIP-seq (2i tracks) and CUT&RUN (Asynchronous, 20 h Thymidine, and Serum/LIF tracks) at select genomic loci, same as shown in S2 Fig, in the mouse genome. **(A–C)**. H3K27me3 enrichment at Gm36649 (**A**), Cdh23 (**B**), and Cpa1 (**C**). All three genes present relatively low levels of H3K27me3 silencing in asynchronous serum/LIF-grown mESCs in comparison to stronger enrichment in 2i-grown cells. Additionally, the gains observed in serum/LIF-grown cells upon G1 arrest mirror the domains seen in 2i cells with boundaries that extend beyond asynchronous serum/LIF. **(D)**. H3K27me3 enrichment at region around Pla2g2f promoter. Asynchronous serum/LIF-grown mESCs show low enrichment at this loci while 2i-grown cells are observed to possess a H3K27me3 domain at the same loci. Notably, upon G1 arrest in serum/LIF cells, this H3K27me3 domain is recapitulated. The genomic snapshot was created using IGV, setting the midpoint of the data range as the lower cut-off used in calling domains. Thus, data above midpoint (red/orange) would belong to domains, whereas data below midpoint (blue/purple) would be outside domains.
(PDF)

S8 Fig G1 shortening in 2i-grown mESCs leads to global H3K27me3 loss. **(A)**. Immunoblot of three biological replicates for mESCs grown in 2i medium then treated with DMSO or Chiron-124 for 20 h followed by acid extraction of histones. **(B)**. Quantification of H3K27me3 levels normalized to DMSO treatment for biological replicates shown in (**A**). (**C**) shows the successive gates applied to the flow data before the analysis of PI histogram for the flow cytometry analysis of DNA content using propidium iodide fluorescence for HEK293 cells that were treated with DMSO or Chiron-124 for 48 h shown in Fig 5A. Data underlying this figure can be found in S10 Data.
(PDF)

S1 Data Data underlying Fig 1. (XLSX)

S2 Data Data underlying Fig 2. (XLSX)

S3 Data Data underlying Fig 3. (XLSX)

S4 Data Data underlying Fig 4. (XLSX)

S5 Data Data underlying Fig 5. (XLSX)

S6 Data Data underlying Fig 6. (XLSX)

S7 Data Data underlying Fig 7. (XLSX)

S8 Data Data underlying S5 Fig. (XLSX)

S9 Data Data underlying S6 Fig. (XLSX)

S10 Data Data underlying S8 Fig. (XLSX)

S11 Data Data underlying S1 Fig. (XLSX)

## Abbreviations

**:**
CDKN2A: cyclin-dependent kinase inhibitor 2A
DMG: diffuse midline glioma
GO: gene ontology
LIF: leukemia inhibitory factor
MS: mass spectrometry
PCA: principal component analysis
PRC2: polycomb repressive complex 2
mESCs: mouse embryonic stem cell lines
Rb: retinoblastoma protein
