# Supplementary material for: The length of the G1 phase is an essential determinant of H3K27me3 landscapes across diverse cell types
Source: PLoS Biol. 2025 Apr 17;23(4):e3003119. doi: 10.1371/journal.pbio.3003119 (PMC12052206; doi:10.1371/journal.pbio.3003119)
Supplement: S1 Raw Images — (PDF) [file pbio.3003119.s001.pdf]

Figure 4A

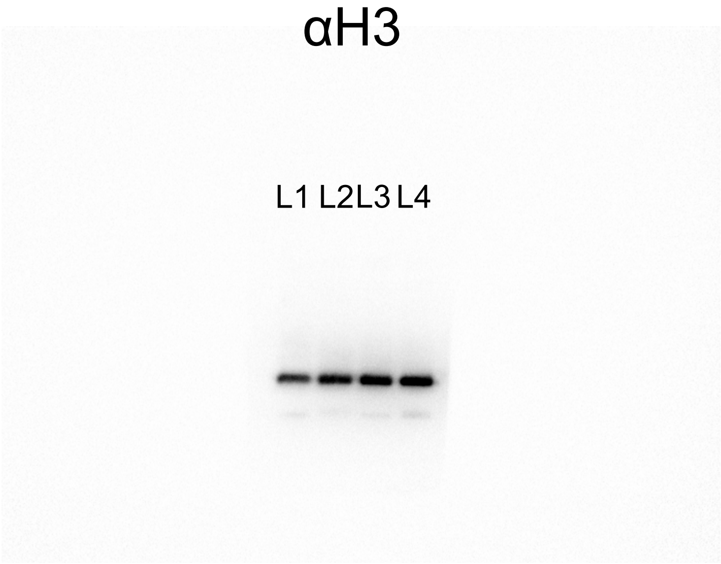

L1 - C6 2i
L2 - C6 Serum/LIF
L3 - 129B-13 2i
L4 - 129B-13 Serum/LIF

Figure 4A

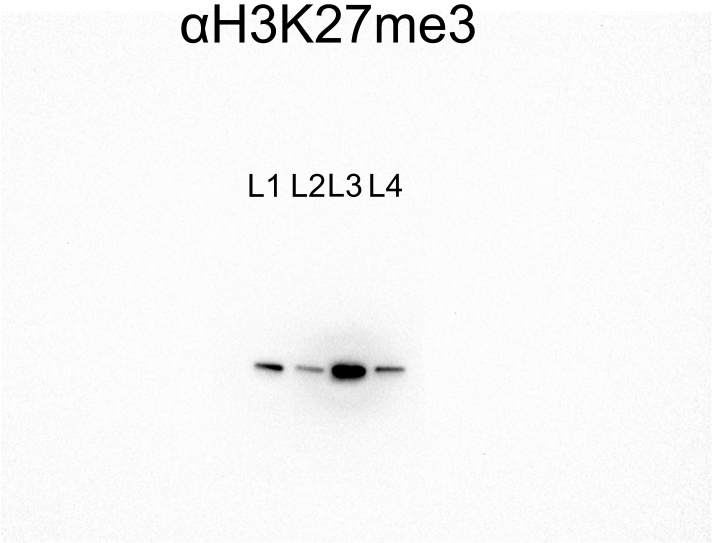

L1 - C6 2i
L2 - C6 Serum/LIF
L3 - 129B-13 2i
L4 - 129B-13 Serum/LIF

Figure 5C

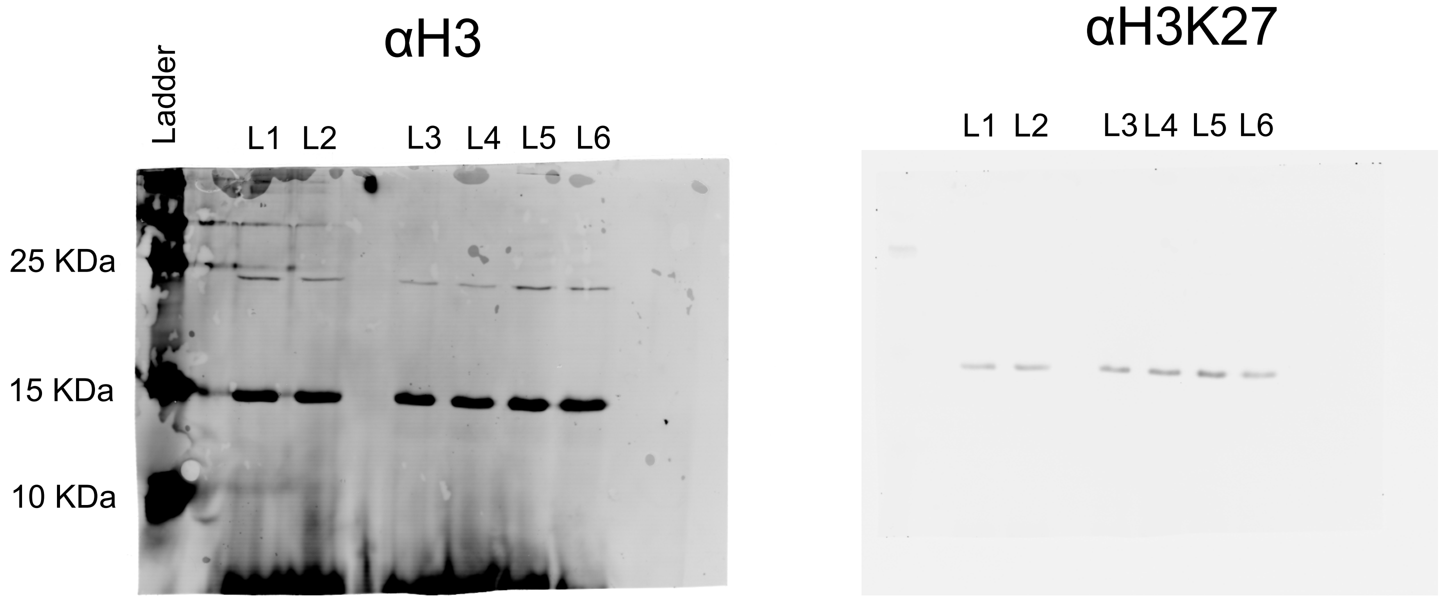

Imaged on Sapphire biomolecular imager with IR secondary antibodies
HEK293 cells

L1 - 24 hour DMSO
L2 - 24 hour Chiron
L3 - 36 hour DMSO
L4 - 36 hour Chiron
L5 - 48 hour DMSO
L6 - 48 hour Chiron

Figure S8

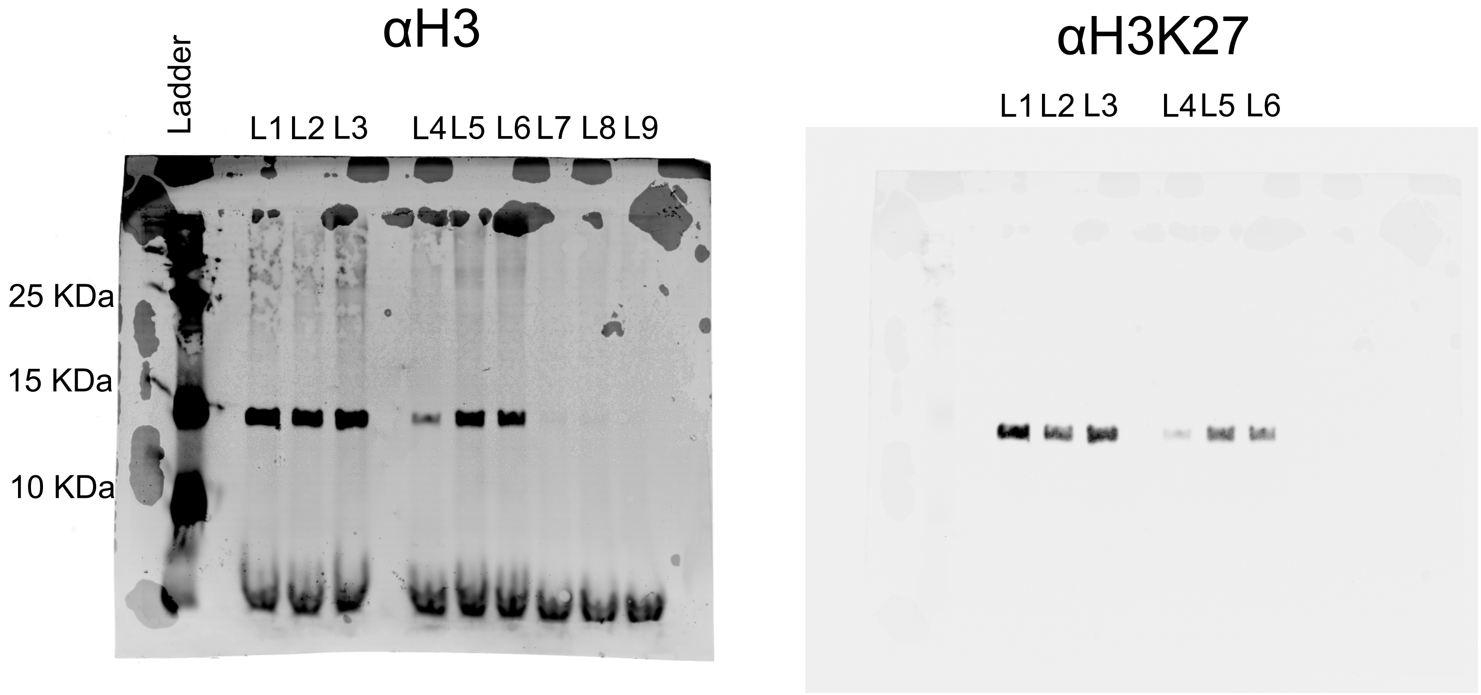

Imaged on Sapphire biomolecular imager with IR secondary antibodies

L1 - mESC 2i DMSO replicate 1
L2 - mESC 2i DMSO replicate 2
L3 - mESC 2i DMSO replicate 3

L4 - mESC 2i Chiron-124 20 hours replicate 1
L5 - mESC 2i Chiron-124 20 hours replicate 2
L6 - mESC 2i Chiron-124 20 hours replicate 3

L7 - mESC 2i Chiron-124 40 hours replicate 1 (protein levels too low to visualize)
L8 - mESC 2i Chiron-124 40 hours replicate 2 (protein levels too low to visualize)
L9 - mESC 2i Chiron-124 40 hours replicate 3 (protein levels too low to visualize)

Figure 6A

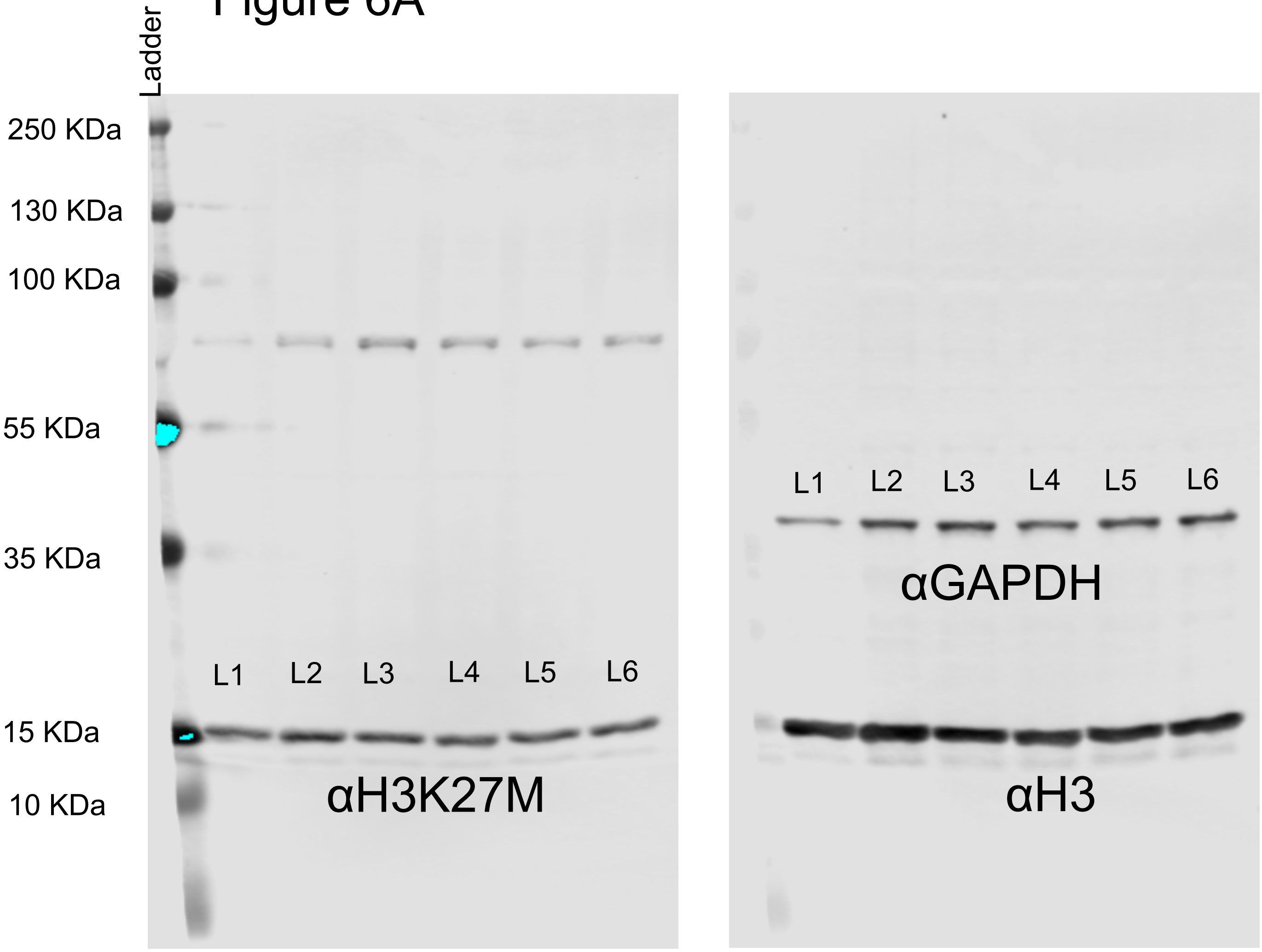

Imaged with IR secondary antibodies

DMG cells

L1 - No treatment
L2 - DMSO
L3 - 1 μM Palbociclib replicate 1
L4 - 1 μM Palbociclib replicate 2
L5 - 10 μM Palbociclib replicate 1
L6 - 10 μM Palbociclib replicate 2

Figure 6A

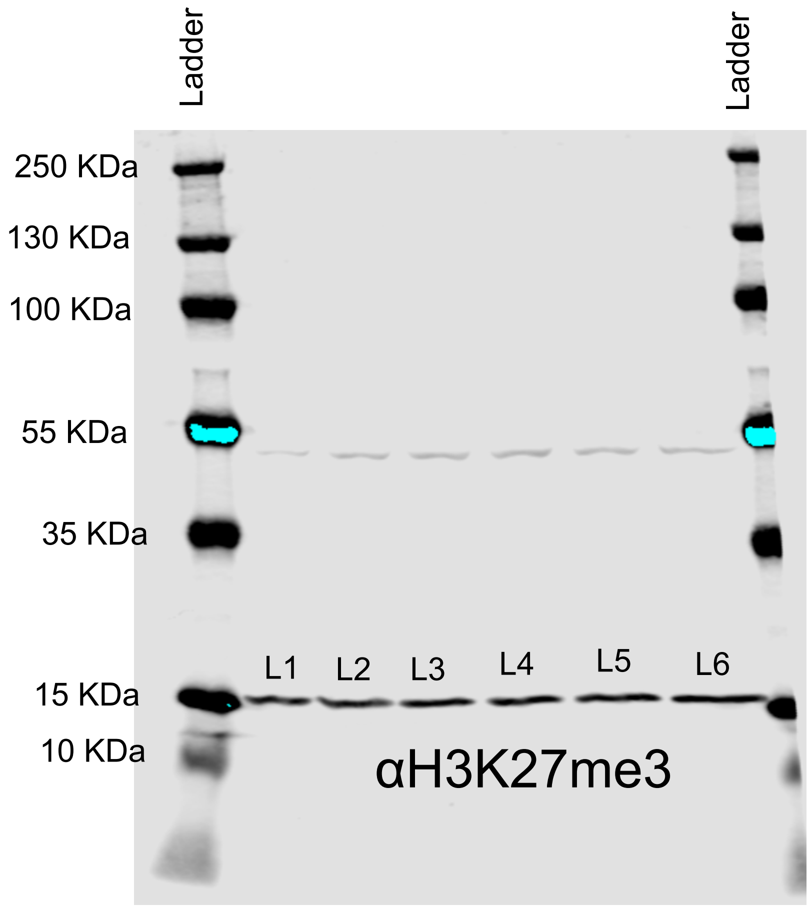

Imaged with IR secondary antibodies

DMG cells

L1 - No treatment
L2 - DMSO
L3 - 1 μM Palbociclib replicate 1
L4 - 1 μM Palbociclib replicate 2
L5 - 10 μM Palbociclib replicate 1
L6 - 10 μM Palbociclib replicate 2
