# Supplementary material for: The length of the G1 phase is an essential determinant of H3K27me3 landscapes across diverse cell types
Source: PLoS Biol. 2025 Apr 17;23(4):e3003119. doi: 10.1371/journal.pbio.3003119 (PMC12052206; doi:10.1371/journal.pbio.3003119)
Supplement: S1 Fig — (A). Flow cytometry analysis of DNA content using propidium iodide fluorescence for asynchronous mESCs grown in serum/LIF medium. (B–J). Same as (A) serum/LIF-grown mESCs treated with the Cdk1 inhibitor RO-3306 for 15 h followed by release from block for 0 h (B), 2 h (C), 4 h (D), 6 h (E), 8 h (F), 10 h (G), 12 h (H), 14 h (I), and 16 h (J). (K). Quantification of percentage of cell population in each phase of the cell cycle (G1, S, and G2) for each release time point profiled in (B) through (J). Analysis shows that upon release from G2 arrest, mESCs take approximately 2–4 h to proceed through the G1 phase and a total cell cycle time of approximately 10 h (compare (B) and (G)). (L), (M) show the successive gates applied to the flow data before the analysis of PI histogram. Data underlying this figure can be found in S11 Data. (PDF) [file pbio.3003119.s002.pdf]

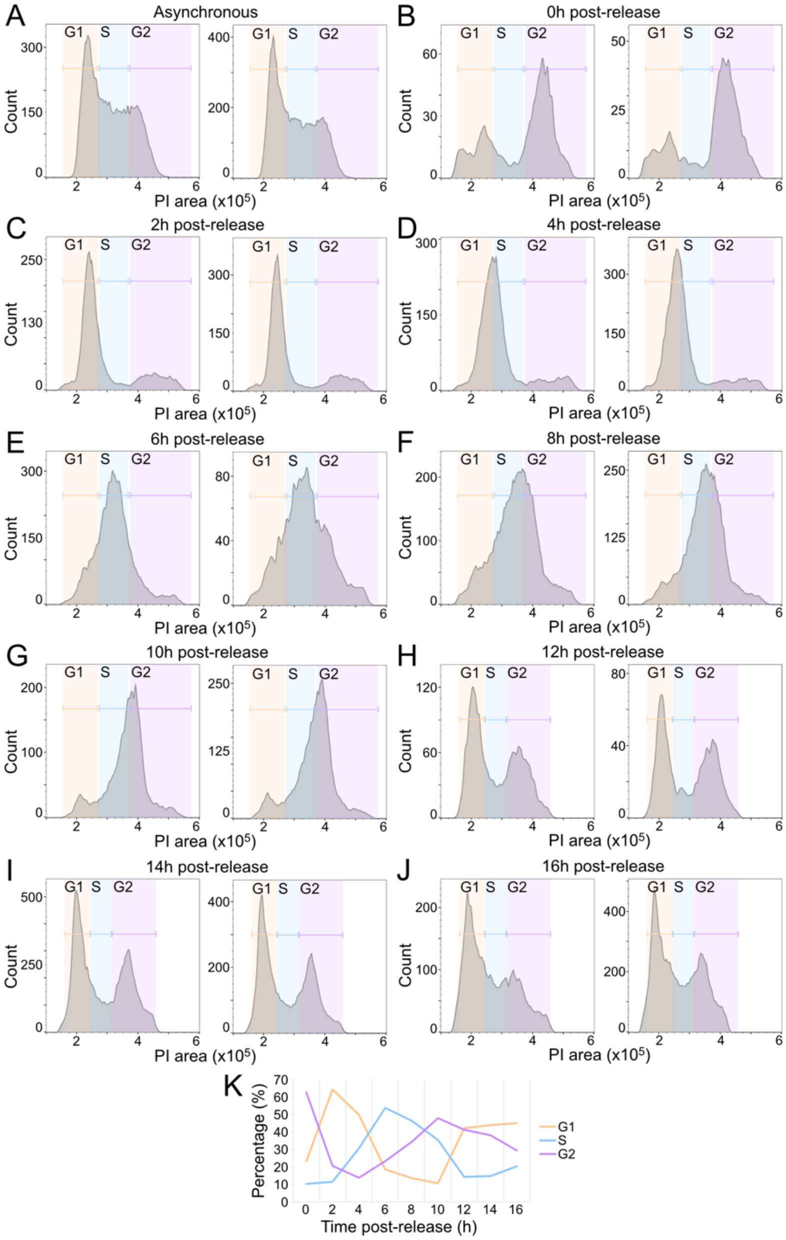
A
Asynchronous
B
0h post-release
C
2h post-release
D
4h post-release
E
6h post-release
F
8h post-release
G
10h post-release
H
12h post-release
I
14h post-release
J
16h post-release
G1
S
G2
Count
PI area (x10^5)
K
Percentage (%)
Time post-release (h)
G1
S
G2

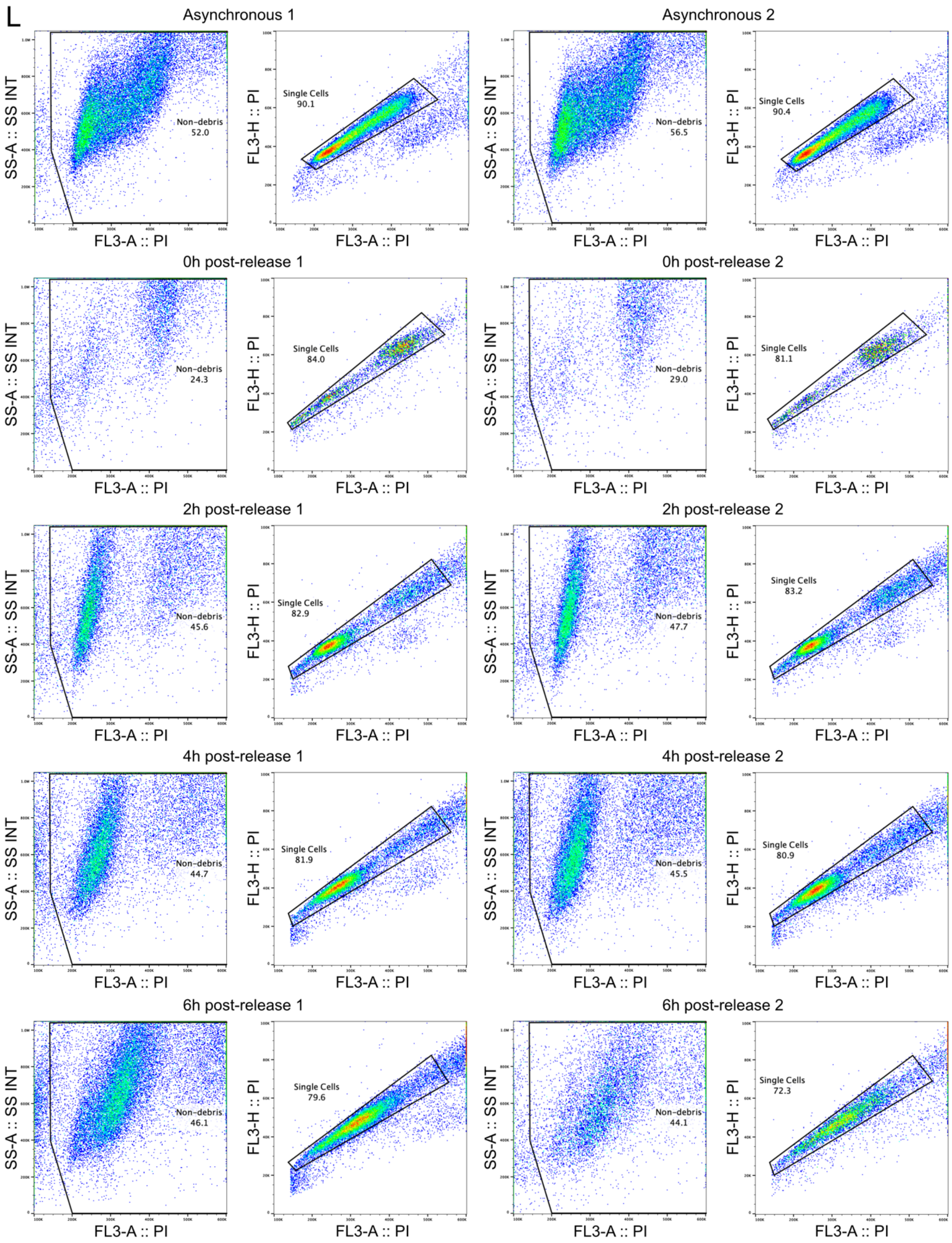
L
Asynchronous 1
Non-debris 52.0
Single Cells 90.1
Asynchronous 2
Non-debris 56.5
Single Cells 90.4
0h post-release 1
Non-debris 24.3
Single Cells 84.0
0h post-release 2
Non-debris 29.0
Single Cells 81.1
2h post-release 1
Non-debris 45.6
Single Cells 82.9
2h post-release 2
Non-debris 47.7
Single Cells 83.2
4h post-release 1
Non-debris 44.7
Single Cells 81.9
4h post-release 2
Non-debris 45.5
Single Cells 80.9
6h post-release 1
Non-debris 46.1
Single Cells 79.6
6h post-release 2
Non-debris 44.1
Single Cells 72.3
SS-A :: SS INT
FL3-H :: PI
FL3-A :: PI

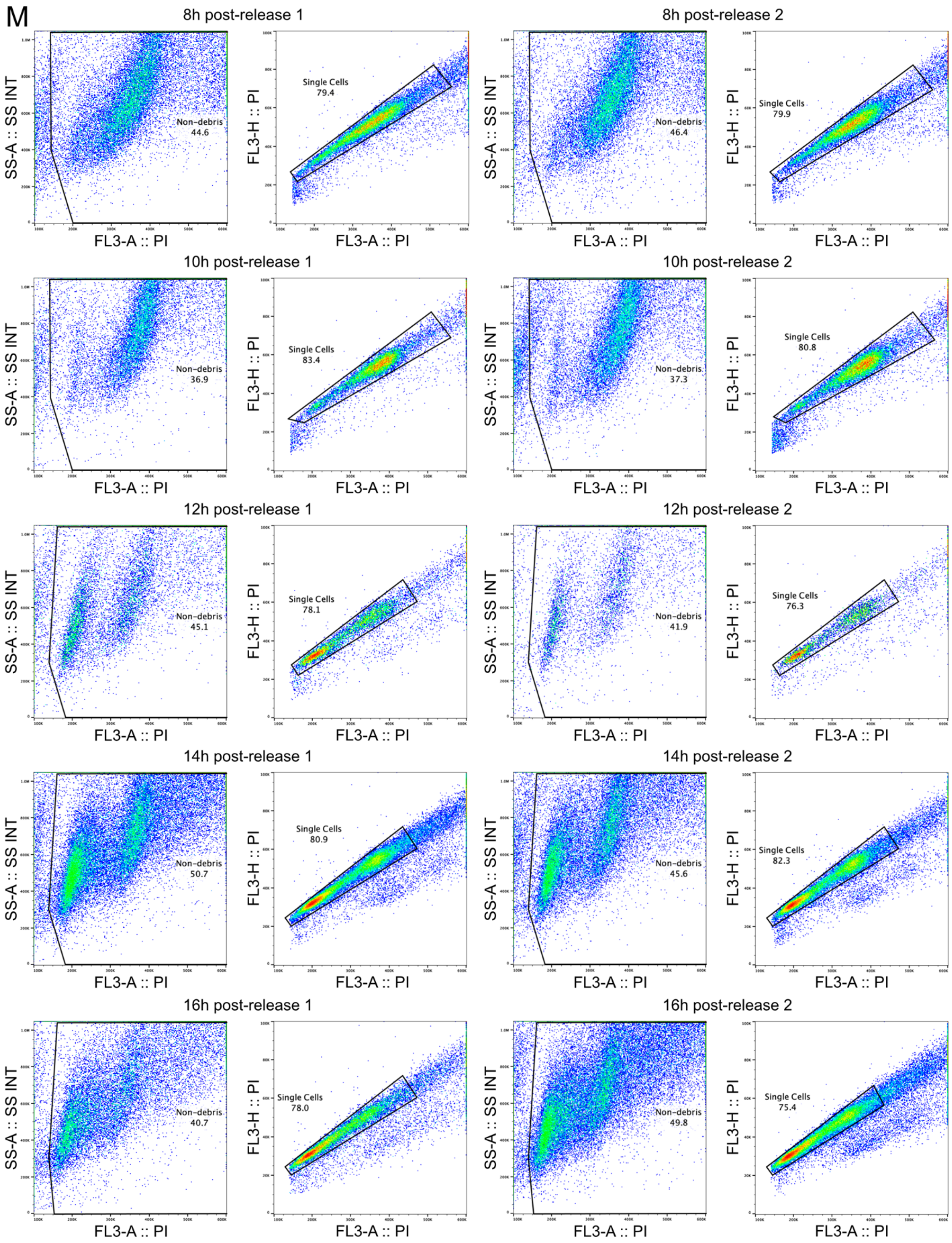

Figure S1. Determination of serum/LIF mESC cell cycle length via G2/M block and release.
A) Flow cytometry analysis of DNA content using propidium iodide fluorescence for asynchronous

mESCs grown in serum/LIF medium. **B-J)** Same as (**A**) serum/LIF-grown mESCs treated with the Cdk1 inhibitor RO-3306 for 15 hours followed by release from block for 0 hours (**B**), 2 hours (**C**), 4 hours (**D**), 6 hours (**E**), 8 hours (**F**), 10 hours (**G**), 12 hours (**H**), 14 hours (**I**), and 16 hours (**J**). **K)** Quantification of percentage of cell population in each phase of the cell cycle (G1, S, and G2) for each release time point profiled in (**B**) through (**J**). Analysis shows that upon release from G2 arrest, mESCs take approximately 2 to 4 hours to proceed through the G1 phase and a total cell cycle time of ~10 hours (compare (**B**) and (**G**)). (**L**), (**M**) show the successive gates applied to the flow data before the analysis of PI histogram. Data underlying this figure can be found in S11 Data.
