# Supplementary material for: The length of the G1 phase is an essential determinant of H3K27me3 landscapes across diverse cell types
Source: PLoS Biol. 2025 Apr 17;23(4):e3003119. doi: 10.1371/journal.pbio.3003119 (PMC12052206; doi:10.1371/journal.pbio.3003119)
Supplement: S2 Fig — Example tracks of normalized H3K27me3 CUT&RUN data at select genomic loci in the mouse genome after G1 arrest for 8, 12, 16, and 20 h via thymidine treatment. (A–C). H3K27me3 enrichment at Gm36649 (A), Cdh23 (B), and Cpa1 (C). These genes present with H3K27me3 domains in asynchronous cells that shows gain both within the existing domain boundaries and spreading past these boundaries, the strength of which is proportional to the length of G1 arrest. (D). H3K27me3 enrichment at region around Pla2g2f. Asynchronous mESCs show little enrichment while progressive G1 arrest leads to the establishment of a stronger H3K27me3 domain. The genomic snapshots were created using IGV, setting the midpoint of the data range as the lower cut-off used in calling domains. Thus, data above midpoint (red) would belong to domains, whereas data below midpoint (blue) would be outside domains. (PDF) [file pbio.3003119.s003.pdf]

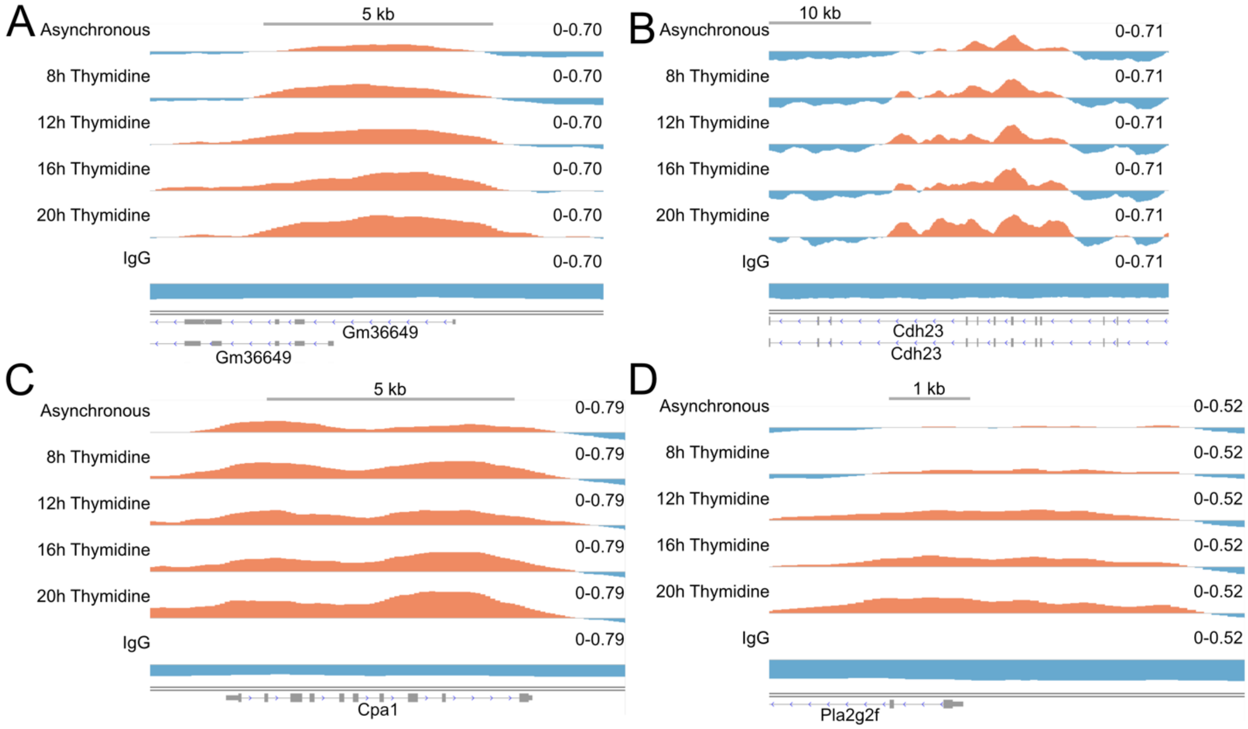

Figure S2. H3K27me3 is gained proportional to length of G1 arrest. Example tracks of normalized H3K27me3 CUT&RUN data at select genomic loci in the mouse genome after G1 arrest for 8, 12, 16, and 20 hours via thymidine treatment. **A-C)** H3K27me3 enrichment at Gm36649 (**A**), Cdh23 (**B**), and Cpa1. These genes present with H3K27me3 domains in asynchronous cells that shows gain both within the existing domain boundaries and spreading past these boundaries, the strength of which is proportional to the length of G1 arrest. **D)** H3K27me3 enrichment at region around Pla2g2f. Asynchronous mESCs show little enrichment while progressive G1 arrest leads to the establishment of a stronger H3K27me3 domain. The genomic snapshots were created using IGV, setting the midpoint of the data range as the lower cut-off used in calling domains. Thus, data above midpoint (red) would belong to domains, whereas data below midpoint (blue) would be outside domains.
