# Supplementary material for: The length of the G1 phase is an essential determinant of H3K27me3 landscapes across diverse cell types
Source: PLoS Biol. 2025 Apr 17;23(4):e3003119. doi: 10.1371/journal.pbio.3003119 (PMC12052206; doi:10.1371/journal.pbio.3003119)
Supplement: S3 Fig — Gene ontology (GO) overrepresentation analysis was performed for genes belonging to clusters 1 and 2, defined from H3K27me3 CUT&RUN data in thymidine-treated serum/LIF-grown mESCs in Fig 2. Unique segments within both clusters lose H3K27me3 enrichment proportional to the length of G1 arrest, with cluster 1 presenting with more loss than cluster 2. GO analysis for both clusters reveals that genes contained in each are enriched for terms related to differentiation and development including cell fate commitment, pattern specification process, and embryonic organ development. (PDF) [file pbio.3003119.s004.pdf]

GO terms enriched for genes in CL1

Gene Set	Description	Size	Expect	Ratio	P Value	↑ FDR
GO:0045165	cell fate commitment	255	16.821	5.0531	<2.2e-16	<2.2e-16
GO:0021953	central nervous system neuron differentiation	196	12.929	4.3312	<2.2e-16	<2.2e-16
GO:0048568	embryonic organ development	472	31.136	3.7898	<2.2e-16	<2.2e-16
GO:0007389	pattern specification process	442	29.157	3.7384	<2.2e-16	<2.2e-16
GO:0043583	ear development	235	15.502	3.5479	<2.2e-16	<2.2e-16
GO:0001228	DNA-binding transcription activator activity, RNA polymerase II-specific	439	28.959	3.2805	<2.2e-16	<2.2e-16
GO:0090596	sensory organ morphogenesis	282	18.602	3.2792	<2.2e-16	<2.2e-16
GO:0001501	skeletal system development	491	32.389	2.9331	<2.2e-16	<2.2e-16
GO:0061564	axon development	458	30.212	2.9127	<2.2e-16	<2.2e-16
GO:0030900	forebrain development	413	27.244	2.8997	<2.2e-16	<2.2e-16
GO:0097060	synaptic membrane	484	31.927	2.7562	<2.2e-16	<2.2e-16
GO:0097485	neuron projection guidance	205	13.523	3.6974	2.2204e-16	2.1385e-14
GO:0060562	epithelial tube morphogenesis	355	23.418	2.9465	2.2204e-16	2.1385e-14
GO:0001655	urogenital system development	356	23.484	2.8530	3.5527e-15	3.1771e-13
GO:0048736	appendage development	190	12.534	3.6702	5.9952e-15	5.0040e-13

GO terms enriched for genes in CL2

Gene Set	Description	Size	Expect	Ratio	P Value	↑ FDR
GO:0098742	cell-cell adhesion via plasma-membrane adhesion molecules	249	22.366	3.3086	<2.2e-16	<2.2e-16
GO:0007389	pattern specification process	442	39.701	2.3929	4.4409e-16	2.7800e-13
GO:0061053	somite development	99	8.8924	3.9360	3.7637e-13	1.5707e-10
GO:0099177	regulation of trans-synaptic signaling	482	43.294	2.0557	2.9788e-11	9.3235e-9
GO:0061564	axon development	458	41.138	2.0662	6.4126e-11	1.6057e-8
GO:0001501	skeletal system development	491	44.103	2.0180	8.2174e-11	1.7147e-8
GO:0060562	epithelial tube morphogenesis	355	31.887	2.1953	2.1098e-10	3.7735e-8
GO:0021510	spinal cord development	106	9.5211	3.3609	4.2400e-10	6.6356e-8
GO:0045165	cell fate commitment	255	22.905	2.4013	6.2789e-10	8.7347e-8
GO:0061448	connective tissue development	278	24.970	2.2827	2.2834e-9	2.8461e-7
GO:0097060	synaptic membrane	484	43.474	1.9322	2.5006e-9	2.8461e-7
GO:0090287	regulation of cellular response to growth factor stimulus	260	23.354	2.3123	3.7401e-9	3.9022e-7
GO:0007626	locomotory behavior	235	21.108	2.3688	6.0891e-9	5.8643e-7
GO:0001525	angiogenesis	488	43.833	1.8935	8.2015e-9	6.8834e-7
GO:0001763	morphogenesis of a branching structure	230	20.659	2.3718	8.2469e-9	6.8834e-7

Figure S3. Gene ontology analysis for regions with H3K27me3 loss upon G1 arrest. Gene ontology (GO) overrepresentation analysis was performed for genes belonging to clusters 1 and 2, defined from H3K27me3 CUT&RUN data in thymidine-treated serum/LIF-grown mESCs in Figure 2. Unique segments within both clusters lose H3K27me3 enrichment proportional to the length of G1 arrest, with cluster 1 presenting with more loss than cluster 2. GO analysis for both clusters reveals that genes contained in each are enriched for terms related to differentiation and development including cell fate commitment, pattern specification process, and embryonic organ development.
