# Supplementary material for: The length of the G1 phase is an essential determinant of H3K27me3 landscapes across diverse cell types
Source: PLoS Biol. 2025 Apr 17;23(4):e3003119. doi: 10.1371/journal.pbio.3003119 (PMC12052206; doi:10.1371/journal.pbio.3003119)
Supplement: S4 Fig — Gene ontology (GO) overrepresentation analysis was performed for genes belonging to clusters 5 and 6, defined from H3K27me3 CUT&RUN data in thymidine-treated serum/LIF-grown mESCs in Fig 2. Unique segments within both clusters gain H3K27me3 enrichment proportional to the length of G1 arrest, with a majority of this gain observed outside of existing H3K27me3 domains in asynchronous cells with cluster 6 presenting with a stronger gain of H3K27me3 and greater proportion of new domains compared to cluster 5. GO analysis for cluster 5 reveals that genes contained in this cluster are enriched for terms related to immune function including adaptive immune response and cytokine receptor binding. GO analysis for cluster 6 reveals that genes contained in this cluster are enriched for terms related to the cytoskeleton and protease activity. (PDF) [file pbio.3003119.s005.pdf]

GO terms enriched for genes in CL5

Gene Set	Description	Size	Expect	Ratio	P Value	↑ FDR
GO:0017171	serine hydrolase activity	216	4.1876	4.5372	4.3845e-8	0.000054894
GO:0002250	adaptive immune response	397	7.6966	3.1183	9.0367e-7	0.00041633
GO:0009897	external side of plasma membrane	371	7.1925	3.1978	9.9760e-7	0.00041633
GO:0045111	intermediate filament cytoskeleton	223	4.3233	3.9322	0.0000017065	0.00053414
GO:0042742	defense response to bacterium	269	5.2151	3.4515	0.0000053917	0.0013501
GO:0005126	cytokine receptor binding	307	5.9518	3.1923	0.0000091107	0.0019011
GO:0030545	receptor regulator activity	490	9.4996	2.6317	0.000010904	0.0019502
GO:0050900	leukocyte migration	315	6.1069	2.9475	0.000045174	0.0070698
GO:0006959	humoral immune response	184	3.5672	3.6443	0.000063151	0.0087850
GO:0038024	cargo receptor activity	83	1.6091	4.9717	0.00020367	0.025499
GO:0042110	T cell activation	458	8.8792	2.3651	0.00024721	0.028137
GO:0030246	carbohydrate binding	275	5.3314	2.8135	0.00031973	0.032617
GO:0070661	leukocyte proliferation	307	5.9518	2.6883	0.00033868	0.032617
GO:0004175	endopeptidase activity	446	8.6466	2.3131	0.00045999	0.041136
GO:0070269	pyroptosis	20	0.38774	10.316	0.00052513	0.043831

GO terms enriched for genes in CL6

Gene Set	Description	Size	Expect	Ratio	P Value	↑ FDR
GO:0045111	intermediate filament cytoskeleton	223	2.5968	5.7764	5.3392e-8	0.000066847
GO:0004175	endopeptidase activity	446	5.1936	3.2733	0.000018666	0.011685
GO:0017171	serine hydrolase activity	216	2.5153	4.3733	0.000046311	0.019327

Figure S4. Gene ontology analysis for regions with H3K27me3 gain upon G1 arrest. Gene ontology (GO) overrepresentation analysis was performed for genes belonging to clusters 5 and 6, defined from H3K27me3 CUT&RUN data in thymidine-treated serum/LIF-grown mESCs in Figure 2. Unique segments within both clusters gain H3K27me3 enrichment proportional to the length of G1 arrest, with a majority of this gain observed outside of existing H3K27me3 domains in asynchronous cells with cluster 6 presenting with a stronger gain of H3K27me3 and greater proportion of new domains compared to cluster 5. GO analysis for cluster 5 reveals that genes contained in this cluster are enriched for terms related to immune function including adaptive immune response and cytokine receptor binding. GO analysis for cluster 6 reveals that genes contained in this cluster are enriched for terms related to the cytoskeleton and protease activity.
