# Supplementary material for: The length of the G1 phase is an essential determinant of H3K27me3 landscapes across diverse cell types
Source: PLoS Biol. 2025 Apr 17;23(4):e3003119. doi: 10.1371/journal.pbio.3003119 (PMC12052206; doi:10.1371/journal.pbio.3003119)
Supplement: S5 Fig — (A). Loadings of the first two principal components from principal component analysis of the three RNA-seq replicates after 20-h G1 arrest via thymidine treatment, along with asynchronous controls. Thymidine-treated samples separate from asynchronous controls while clustering together amongst themselves. (B). Log2 normalized RNA counts of key pluripotency and differentiation markers from the RNA-seq replicates shown in (A). Expression of the pluripotency markers Nanog and Oct4 are high in both thymidine-treated and asynchronous samples, while differentiation markers remain low in both conditions. Data underlying this figure can be found in S8 Data. (PDF) [file pbio.3003119.s006.pdf]

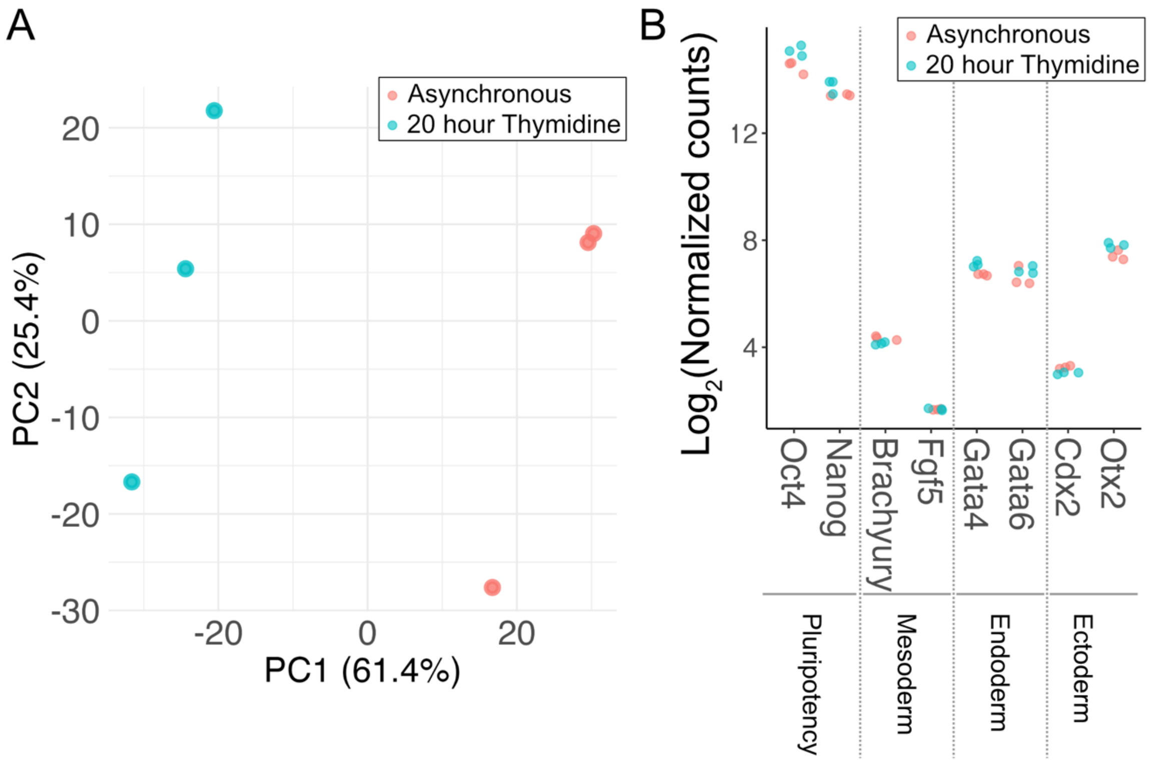

Figure S5. G1 arrest via thymidine treatment in mESCs shows reproducibility while maintaining pluripotency. A) Loadings of the first two principal components from principal component analysis of the three RNA-seq replicates after 20-hour G1 arrest via thymidine treatment, along with asynchronous controls. Thymidine-treated samples separate from asynchronous controls while clustering together amongst themselves. **B)** Log_2 normalized RNA counts of key pluripotency and differentiation markers from the RNA-seq replicates shown in (**A**). Expression of the pluripotency markers *Nanog* and *Oct4* are high in both thymidine-treated and asynchronous samples, while differentiation markers remain low in both conditions. Data underlying this figure can be found in S8 Data.
