# Supplementary material for: The length of the G1 phase is an essential determinant of H3K27me3 landscapes across diverse cell types
Source: PLoS Biol. 2025 Apr 17;23(4):e3003119. doi: 10.1371/journal.pbio.3003119 (PMC12052206; doi:10.1371/journal.pbio.3003119)
Supplement: S6 Fig — Plot of log2 ratio of H3K27me3 levels in thymidine-treated compared to asynchronous serum-grown mESCs for unique domain segments as measured by CUT&Tag (x-axis) versus CUT&RUN (y-axis) experiments. Hexagonal binning was performed using geom_hex in ggplot2 and a linear model fit is shown as a red line. Pearson correlation coefficient and the corresponding p-value for the comparison between the two datasets is shown on the top left corner of the plot. Data underlying this figure can be found in S9 Data. (PDF) [file pbio.3003119.s007.pdf]

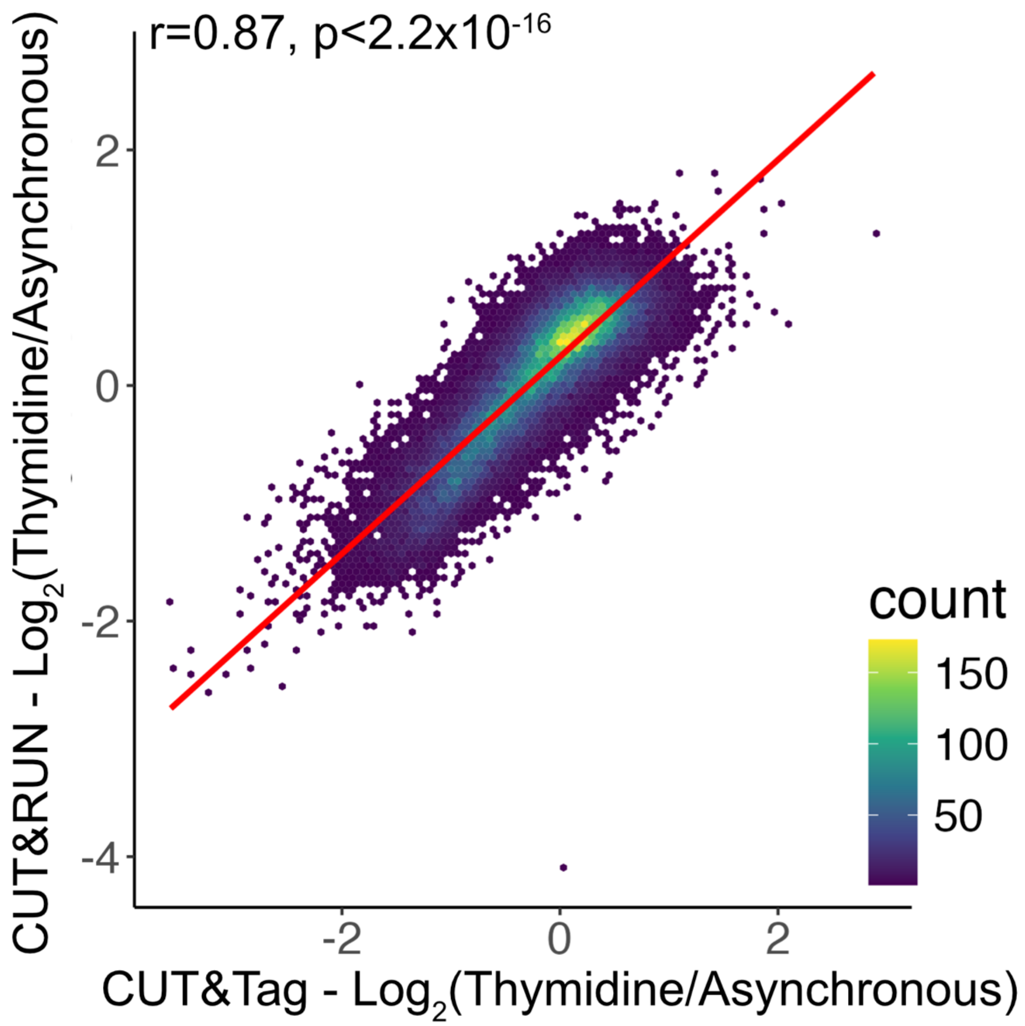

Figure S6. Correlation between H3K27me3 enrichment changes captured by CUT&RUN versus CUT&Tag. Plot of $\log_2$ ratio of H3K27me3 levels in thymidine-treated compared to asynchronous serum-grown mESCs for unique domain segments as measured by CUT&Tag (x-axis) versus CUT&RUN (y-axis) experiments. Hexagonal binning was performed using geom_hex in ggplot2 and a linear model fit is shown as a red line. Pearson correlation coefficient and the corresponding p-value for the comparison between the two datasets is shown on the top left corner of the plot. Data underlying this figure can be found in S9 Data.
