# Supplementary material for: The length of the G1 phase is an essential determinant of H3K27me3 landscapes across diverse cell types
Source: PLoS Biol. 2025 Apr 17;23(4):e3003119. doi: 10.1371/journal.pbio.3003119 (PMC12052206; doi:10.1371/journal.pbio.3003119)
Supplement: S7 Fig — Example tracks of normalized H3K27me3 ChIP-seq (2i tracks) and CUT&RUN (Asynchronous, 20 h Thymidine, and Serum/LIF tracks) at select genomic loci, same as shown in S2 Fig, in the mouse genome. (A–C). H3K27me3 enrichment at Gm36649 (A), Cdh23 (B), and Cpa1 (C). All three genes present relatively low levels of H3K27me3 silencing in asynchronous serum/LIF-grown mESCs in comparison to stronger enrichment in 2i-grown cells. Additionally, the gains observed in serum/LIF-grown cells upon G1 arrest mirror the domains seen in 2i cells with boundaries that extend beyond asynchronous serum/LIF. (D). H3K27me3 enrichment at region around Pla2g2f promoter. Asynchronous serum/LIF-grown mESCs show low enrichment at this loci while 2i-grown cells are observed to possess a H3K27me3 domain at the same loci. Notably, upon G1 arrest in serum/LIF cells, this H3K27me3 domain is recapitulated. The genomic snapshot was created using IGV, setting the midpoint of the data range as the lower cut-off used in calling domains. Thus, data above midpoint (red/orange) would belong to domains, whereas data below midpoint (blue/purple) would be outside domains. (PDF) [file pbio.3003119.s008.pdf]

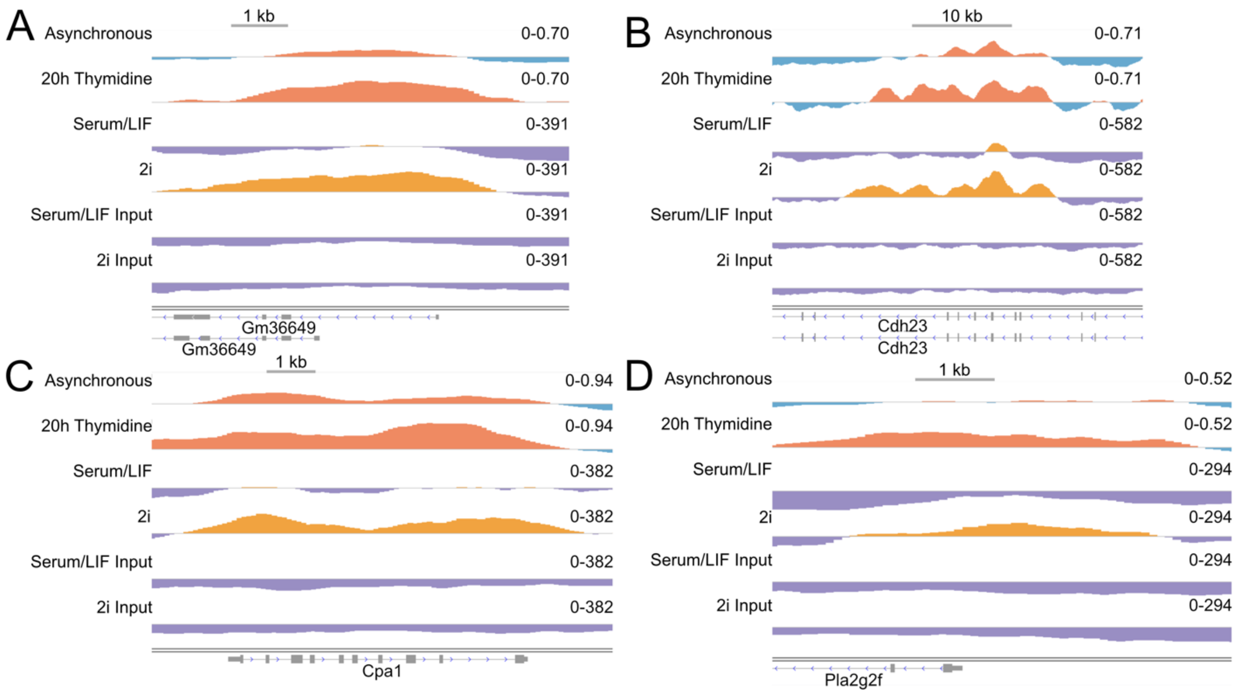

Figure S7. H3K27me3-enriched domains in 2i mESCs reflect gains seen upon G1 arrest in serum-grown cells. Example tracks of normalized H3K27me3 ChIP-seq (2i tracks) and CUT&RUN (Asynchronous, 20h Thymidine, and Serum/LIF tracks) at select genomic loci, same as shown in **Figure S2**, in the mouse genome. **A-C)** H3K27me3 enrichment at Gm36649 (**A**), Cdh23 (**B**), and Cpa1 (**C**). All three genes present relatively low levels of H3K27me3 silencing in asynchronous serum/LIF-grown mESCs in comparison to stronger enrichment in 2i-grown cells. Additionally, the gains observed in serum/LIF-grown cells upon G1 arrest mirror the domains seen in 2i cells with boundaries that extend beyond asynchronous serum/LIF. **D)** H3K27me3 enrichment at region around Pla2g2f promoter. Asynchronous serum/LIF-grown mESCs show low enrichment at this loci while 2i-grown cells are observed to possess a H3K27me3 domain at the same loci. Notably, upon G1 arrest in serum/LIF cells, this H3K27me3 domain is recapitulated. The genomic snapshot was created using IGV, setting the midpoint of the data range as the lower cut-off used in calling domains. Thus, data above midpoint (red/orange) would belong to domains, whereas data below midpoint (blue/purple) would be outside domains.
