# Supplementary material for: The length of the G1 phase is an essential determinant of H3K27me3 landscapes across diverse cell types
Source: PLoS Biol. 2025 Apr 17;23(4):e3003119. doi: 10.1371/journal.pbio.3003119 (PMC12052206; doi:10.1371/journal.pbio.3003119)
Supplement: S8 Fig — (A). Immunoblot of three biological replicates for mESCs grown in 2i medium then treated with DMSO or Chiron-124 for 20 h followed by acid extraction of histones. (B). Quantification of H3K27me3 levels normalized to DMSO treatment for biological replicates shown in (A). (C) shows the successive gates applied to the flow data before the analysis of PI histogram for the flow cytometry analysis of DNA content using propidium iodide fluorescence for HEK293 cells that were treated with DMSO or Chiron-124 for 48 h shown in Fig 5A. Data underlying this figure can be found in S10 Data. (PDF) [file pbio.3003119.s009.pdf]

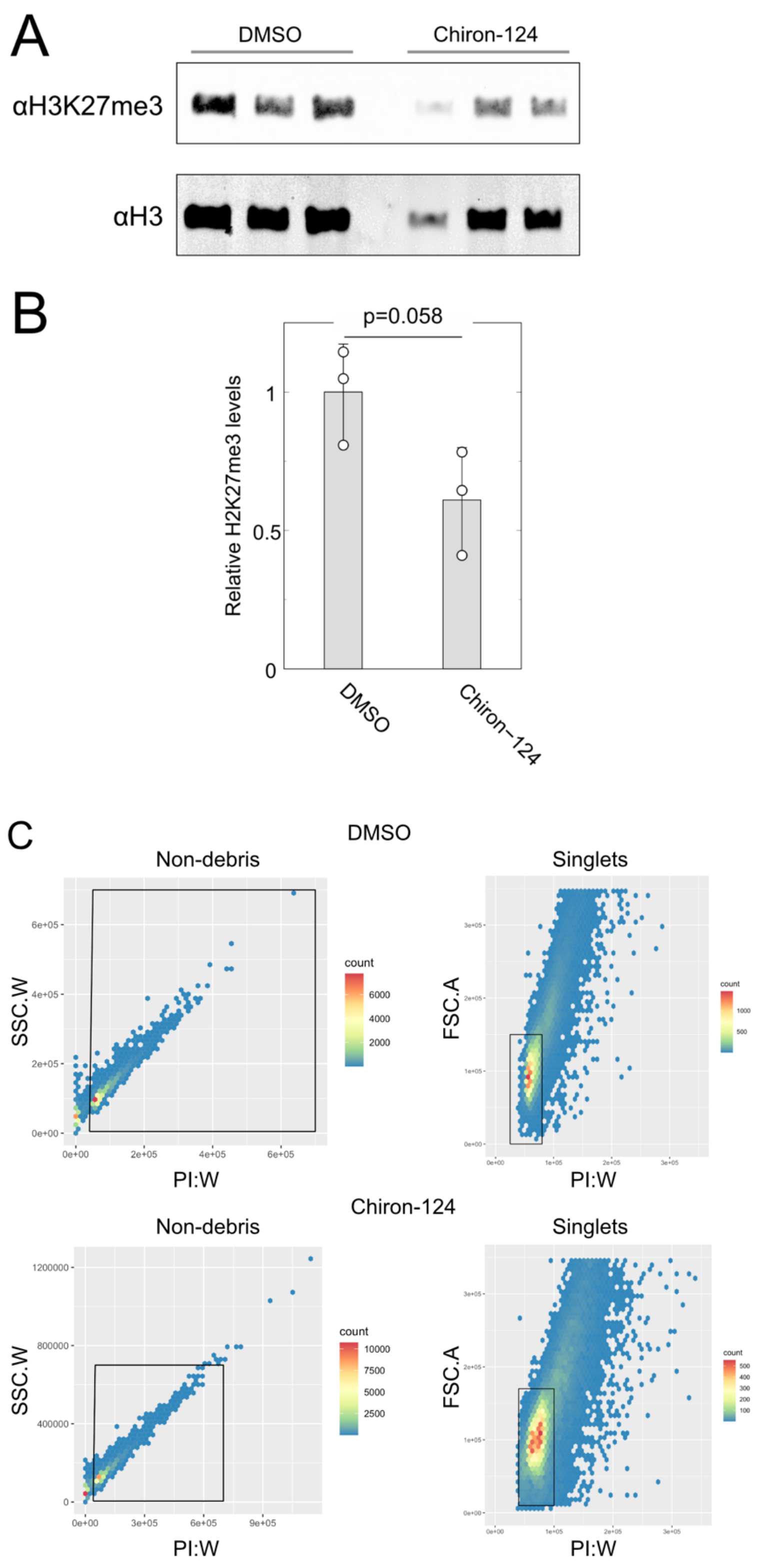

Figure S8. G1 shortening in 2i-grown mESCs leads to global H3K27me3 loss. A) Immunoblot of three biological replicates for mESCs grown in 2i medium then treated with DMSO or Chiron-

124 for 20 hours followed by acid extraction of histones. **B)** Quantification of H3K27me3 levels normalized to DMSO treatment for biological replicates shown in (**A**). (**C**) shows the successive gates applied to the flow data before the analysis of PI histogram for the flow cytometry analysis of DNA content using propidium iodide fluorescence for HEK293 cells that were treated with DMSO for 48 hours shown in Figure 5A.
